# Supplementary figures and images for: Disrupting phage liquid crystalline droplets restores antibiotic susceptibility in Pseudomonas aeruginosa biofilms
Source: PLoS Biol. 2026 Jun 5;24(6):e3003834. doi: 10.1371/journal.pbio.3003834 (PMC13262939; doi:10.1371/journal.pbio.3003834)

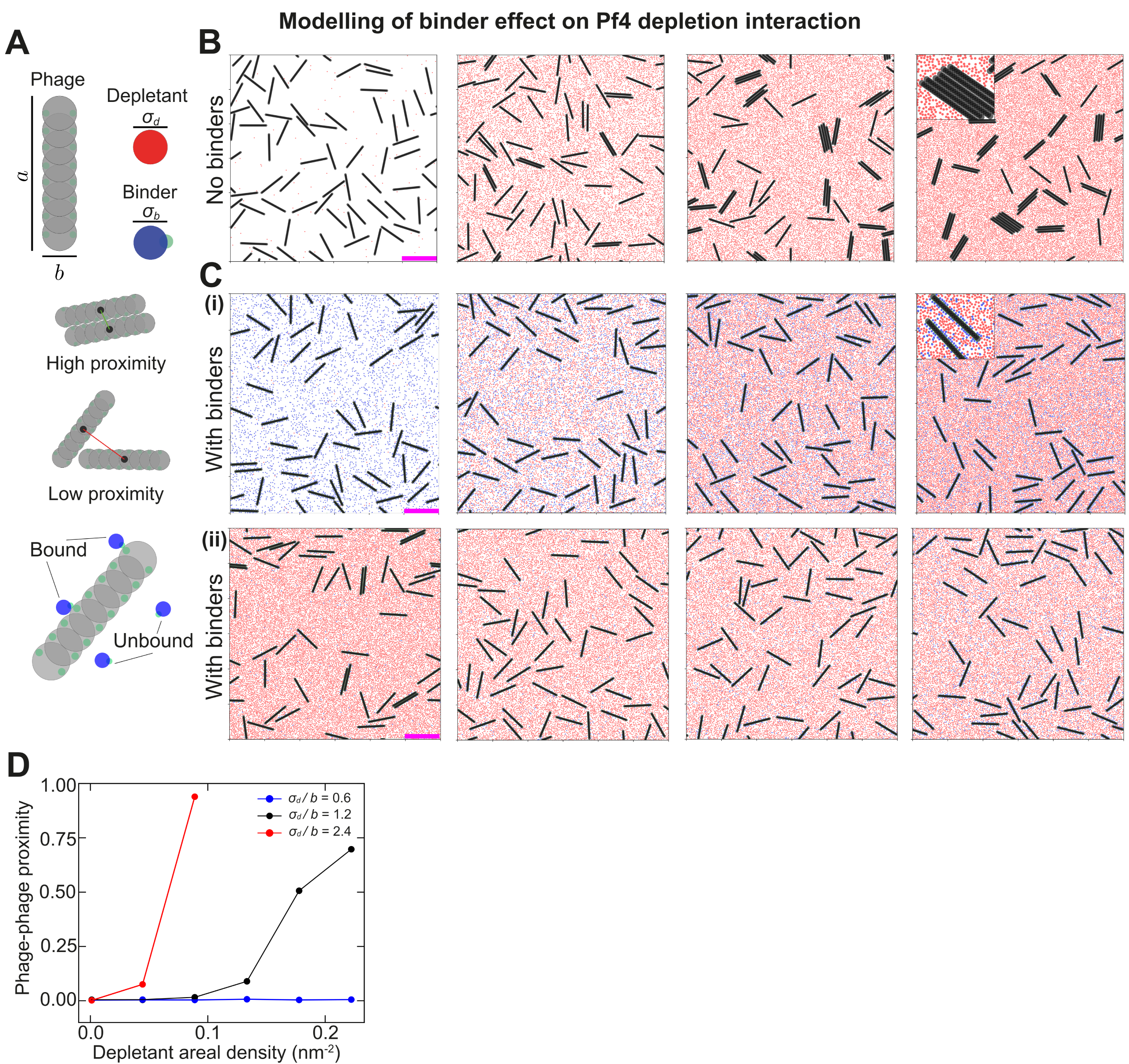

Supplement: S1 Fig — (A) Components of the coarse-grained molecular dynamics model (top): phages are modeled as hard rods of length a = 80.0 nm, and width b = 6.0 nm, the depletant particles are modeled as hard discs with a diameter sd = 2.4 nm and the binders are modeled as hard discs of diameter sb = 2.4 nm with a single (fixed) circular binding patch that is only attracted to the circular binding patches on the phage rod. Visual depiction of phage-phage alignment and localization, here termed proximity (quantified below). (B) Simulation snapshots of 65 phages with increasing numbers of depletant particles (100, 8,000, 16,000, 20,000) from left to right, phages show greater proximity for increased numbers of depletant particles. Scale bar is 100 nm. (C) The presence of binders significantly disrupts depletion attraction between rods. (i) Same as (B) but with a constant presence of 5,000 binders. Despite the increase in depletant numbers, phage proximity remains low. (ii) Increasing the number of binders (400, 625, 1,250, and 2,500) from left to right with 20,000 depletant particles. Even a small number of binders disrupts the depletion interaction between rods. (D) Quantification of phage-phage proximity, the number of aligned phage pairs whose centers are within an approximate phage width divided by twice the number of phages, as a function of depletant density. Lines show different ratios of the depletant diameter and phage width. In panel D, error bars on plots from averaging over multiple time points and simulations are too small to be visible. The data underlying this Figure can be found in S1 Data. (TIF) [file pbio.3003834.s001.tif]

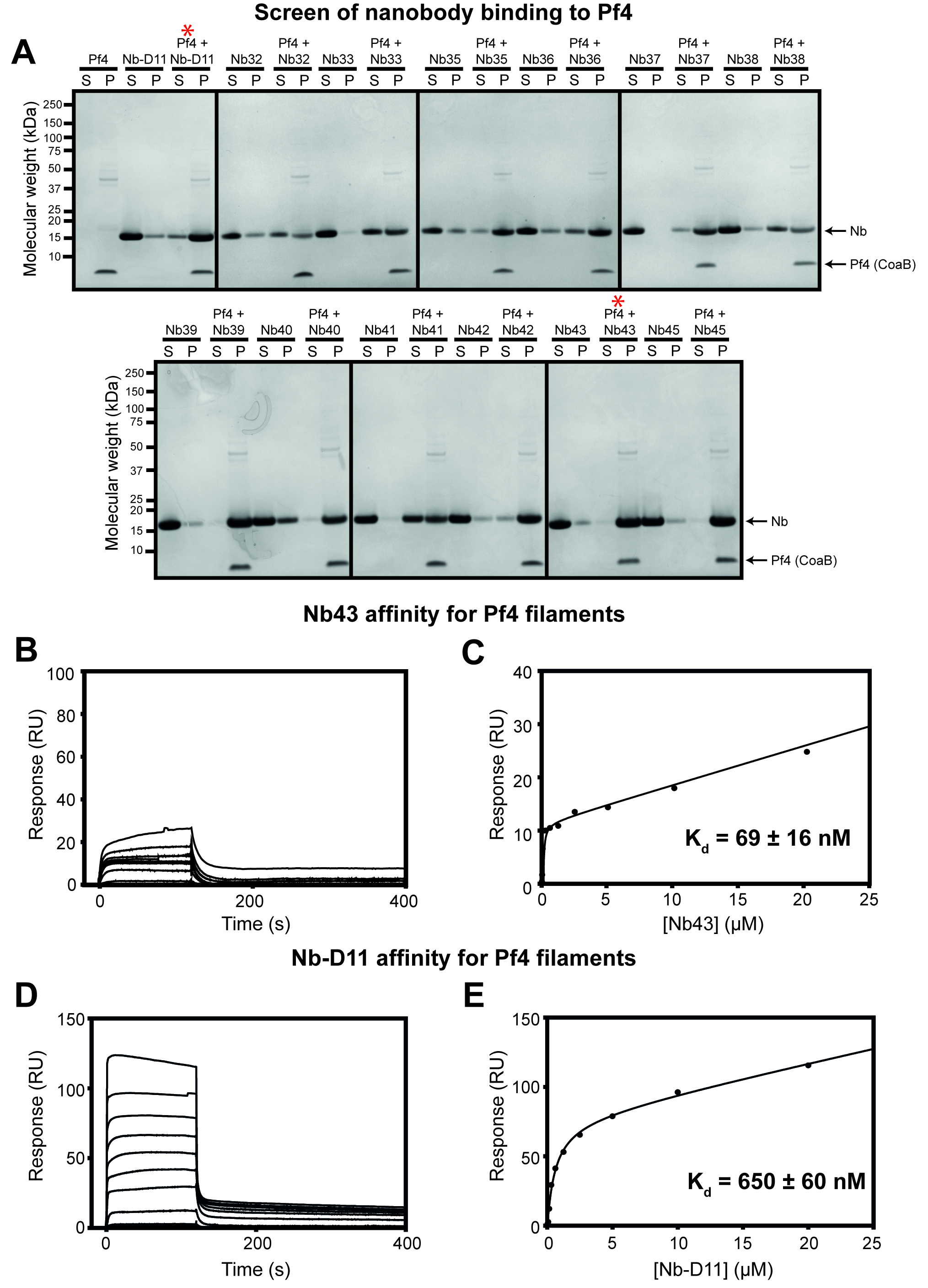

Supplement: S2 Fig — (A) Coomassie-stained SDS-PAGE of the soluble (S) and pellet (P) fractions from Pf4 filament co-sedimentation assay with a panel of recombinant nanobodies. Nanobodies (1 μg) were incubated alone or in the presence of Pf4 (1 μg) as indicated prior to centrifugation at 100,000 g. Without Pf4, nanobodies remained predominantly in the soluble fraction, but nanobodies incubated with Pf4 co-sedimented with Pf4 in the pellet fraction after centrifugation, indicating binding to Pf4. Arrows indicate bands corresponding to nanobody (Nb) and the Pf4 major coat protein (CoaB). Molecular weight markers are shown on the left. Red asterisks denote nanobodies selected for further characterization (Nb43 and Nb-D11). (B–E) Pf4 was immobilized via amide coupling onto a C1-sensor chip and nanobodies were flowed over the chip. (B) Nb43 sensorgram and (C) response curve showing Nb43 has a low nanomolar affinity for Pf4 (Kd = 69 ± 16 nM), (D) Nb-D11 sensorgram and (E) response curve showing Nb-D11 has a high nanomolar affinity for Pf4 (Kd = 650 ± 60 nM). The data underlying this Figure can be found in S1 Data and S1 Raw Images. (TIF) [file pbio.3003834.s002.tif]

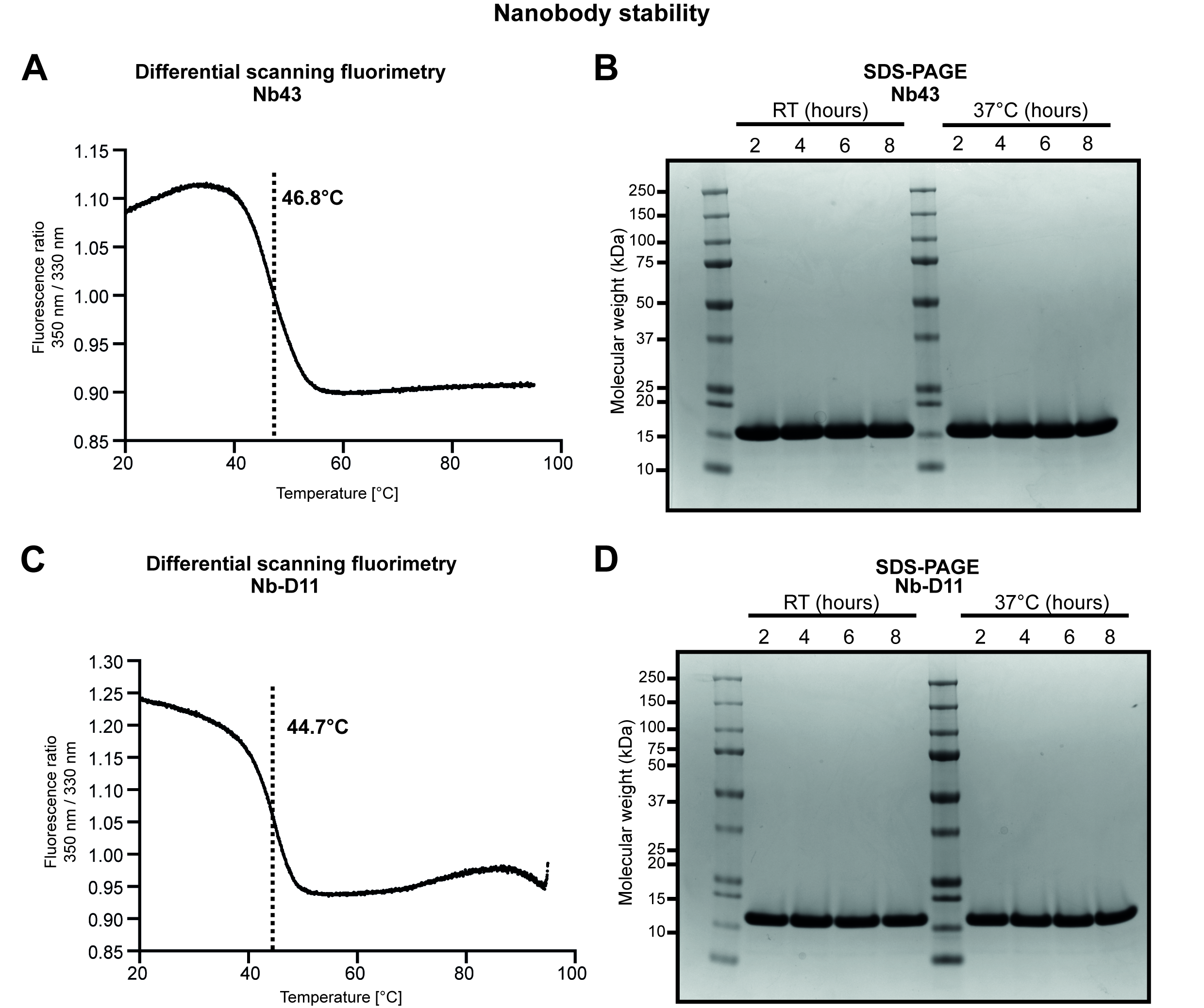

Supplement: S3 Fig — (A) Differential scanning fluorimetry of Nb43. Melting curve for 1 µM Nb43 over a temperature range of 20–95 °C. Intrinsic fluorescence was measured as the ratio of fluorescence at 350 nm/ 330 nm. Nb43 has a Tm of 46.8 °C.(B) Time-course of Nb43 stability at room temperature (RT) and 37 °C. Nb43 was incubated at the indicated temperature for the indicated time before analysis by SDS-PAGE to assay for protein degradation. (C) Differential scanning fluorimetry of Nb-D11. Melting curve for 1 µM Nb-D11 over a temperature range of 20–95 °C. Intrinsic fluorescence was measured as the ratio of fluorescence at 350 nm/ 330 nm. Nb-D11 has a Tm of 44.7 °C. (D) Time-course of Nb-D11 stability at RT and 37 °C. Nb43 was incubated at the indicated temperature for the indicated time before analysis by SDS-PAGE to assay for protein degradation. The data underlying this Figure can be found in S1 Data and S1 Raw Images. (TIF) [file pbio.3003834.s003.tif]

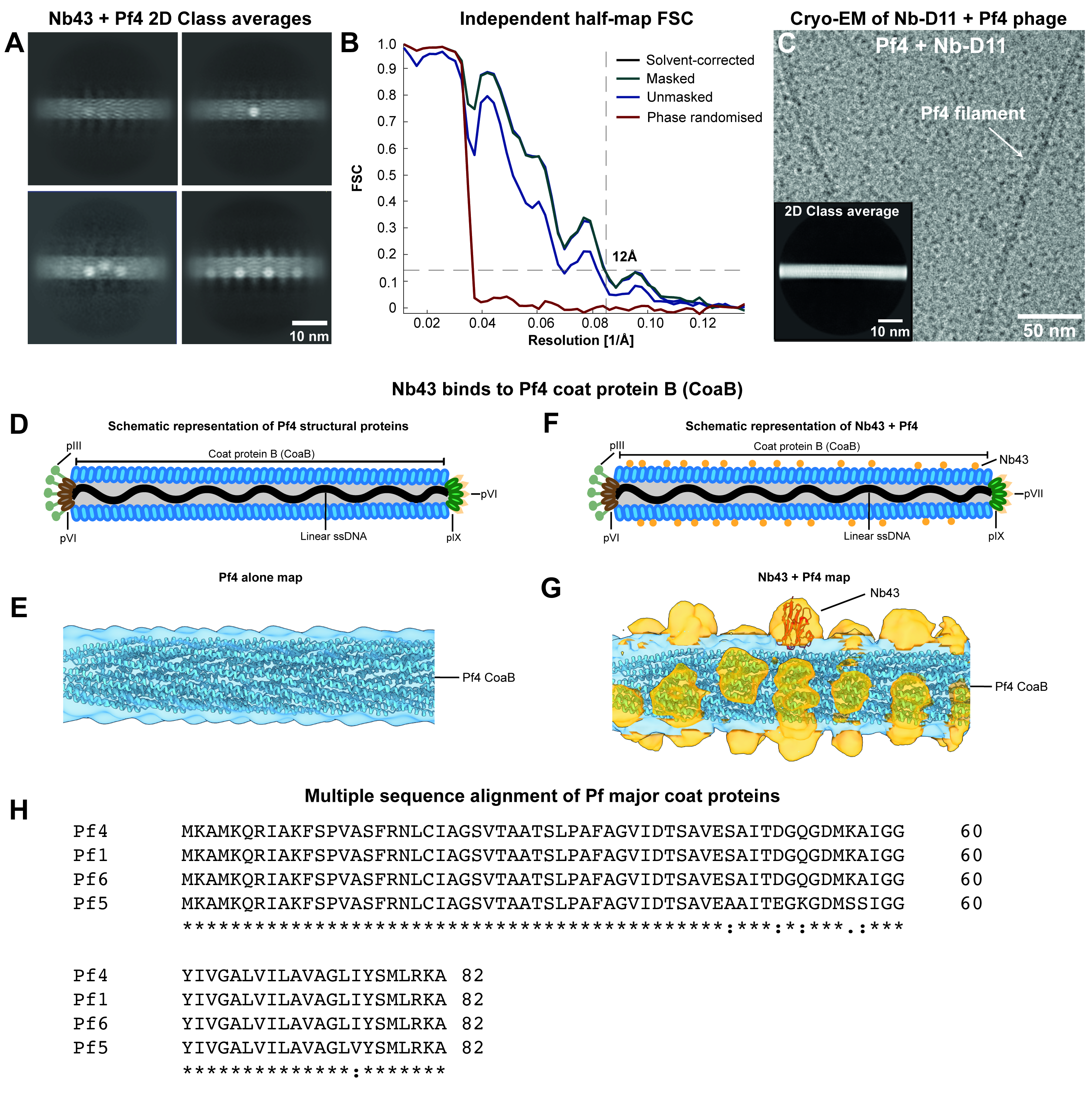

Supplement: S4 Fig — (A) Gallery of 2D class averages illustrating heterogeneity of Nb43 occupancy on Pf4 filaments. (B) Resolution estimation of Pf4-Nb43 reconstruction (Fig 1I) by Fourier shell correlation (FSC) of independently aligned and averaged half-maps. The dashed line indicates 0.143 criterion. (C) Cryo-EM micrograph of Pf4 with Nb-D11, showing Pf4 filaments with no apparent decoration. The inset shows a representative 2D class average. (D) Schematic representation of Pf4 phage showing distribution of Pf4 structural proteins. (E) Cryo-EM density of Pf4 alone (EMDB-10593, low pass filtered to 15 Å) with model fitted in blue. (F) Schematic representation of Pf4 phage showing distribution of Pf4 structural proteins bound to Nb43. (G) Map of Pf4-Nb43 with density corresponding to the Pf4 filament coloured blue, and Nb43 density coloured orange. Nb43 density is seen decorating the surface of Pf4 phage showing direct binding to Pf4 CoaB, the sole protein present along the phage filament surface. (H) Clustal Omega multiple sequence alignment of CoaB proteins from different Pf phages. * = sequence identity, : = strong sequence homology, . = weak sequence homology. The data underlying this Figure can be found in S1 Data. (TIF) [file pbio.3003834.s004.tif]

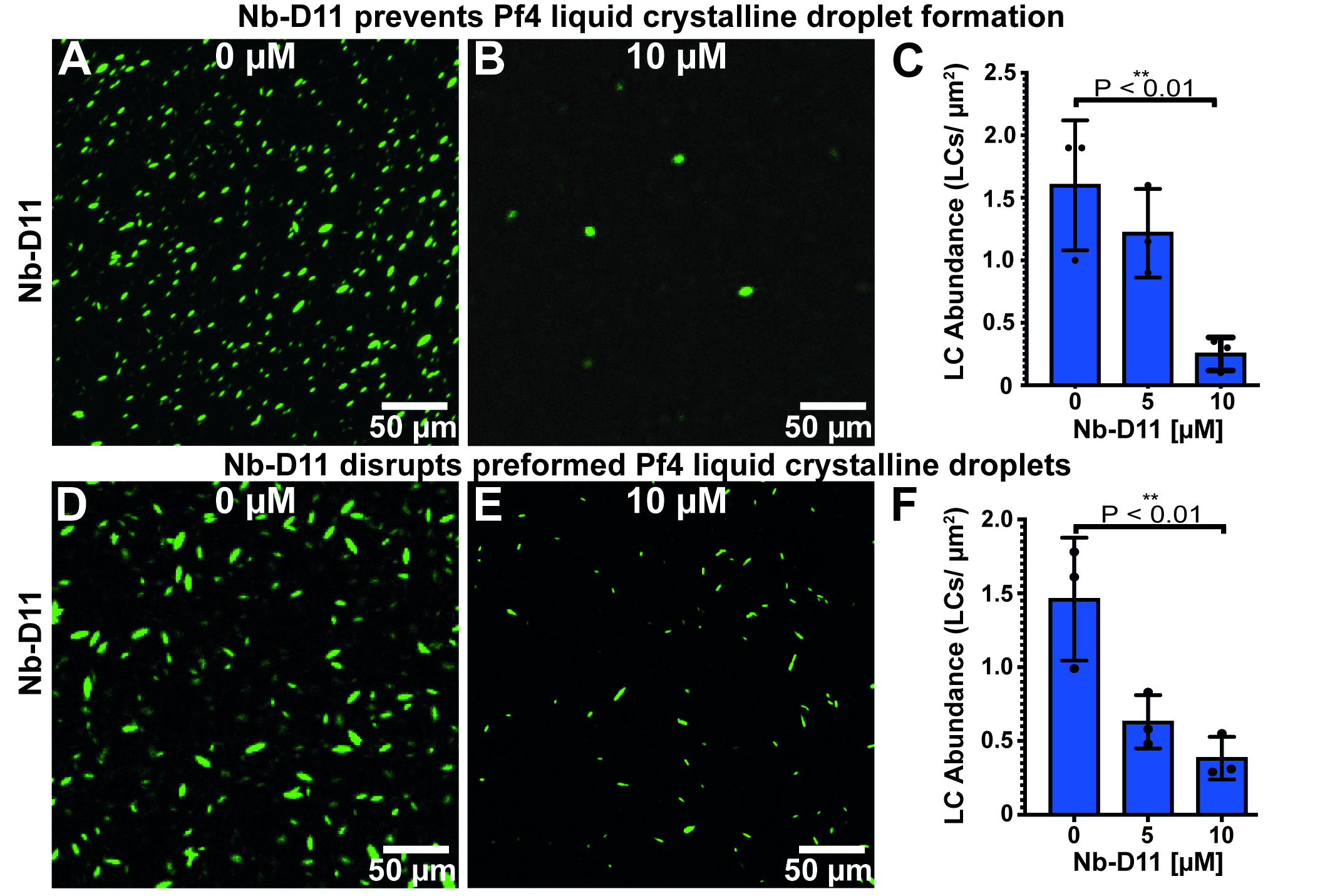

Supplement: S5 Fig — (A and B) Representative light microscopy images of A488-labeled Pf4 liquid crystalline droplets formed in the presence of (A) no Nb-D11 or (B) 10 μM Nb-D11. (C) Bar chart showing the abundance of Pf4 liquid crystalline droplets per μm2. Addition of 10 μM Nb-D11 results in a statistically significant reduction in liquid crystalline droplet formation (Pvalue < 0.01). (D and E) Representative light microscopy images of preformed liquid crystalline droplets treated with (D) no Nb-D11 or (E) 10 μM Nb-D11. (F) Bar chart showing the abundance of Pf4 liquid crystalline droplets per μm2 after treatment. Addition of 10 μM Nb-D11 results in a statistically significant reduction in droplet abundance (Pvalue < 0.01). Error bars represent standard deviation. P-values were calculated using an unpaired t test. All images have been background subtracted. The data underlying this Figure can be found in S1 Data. (TIF) [file pbio.3003834.s005.tif]

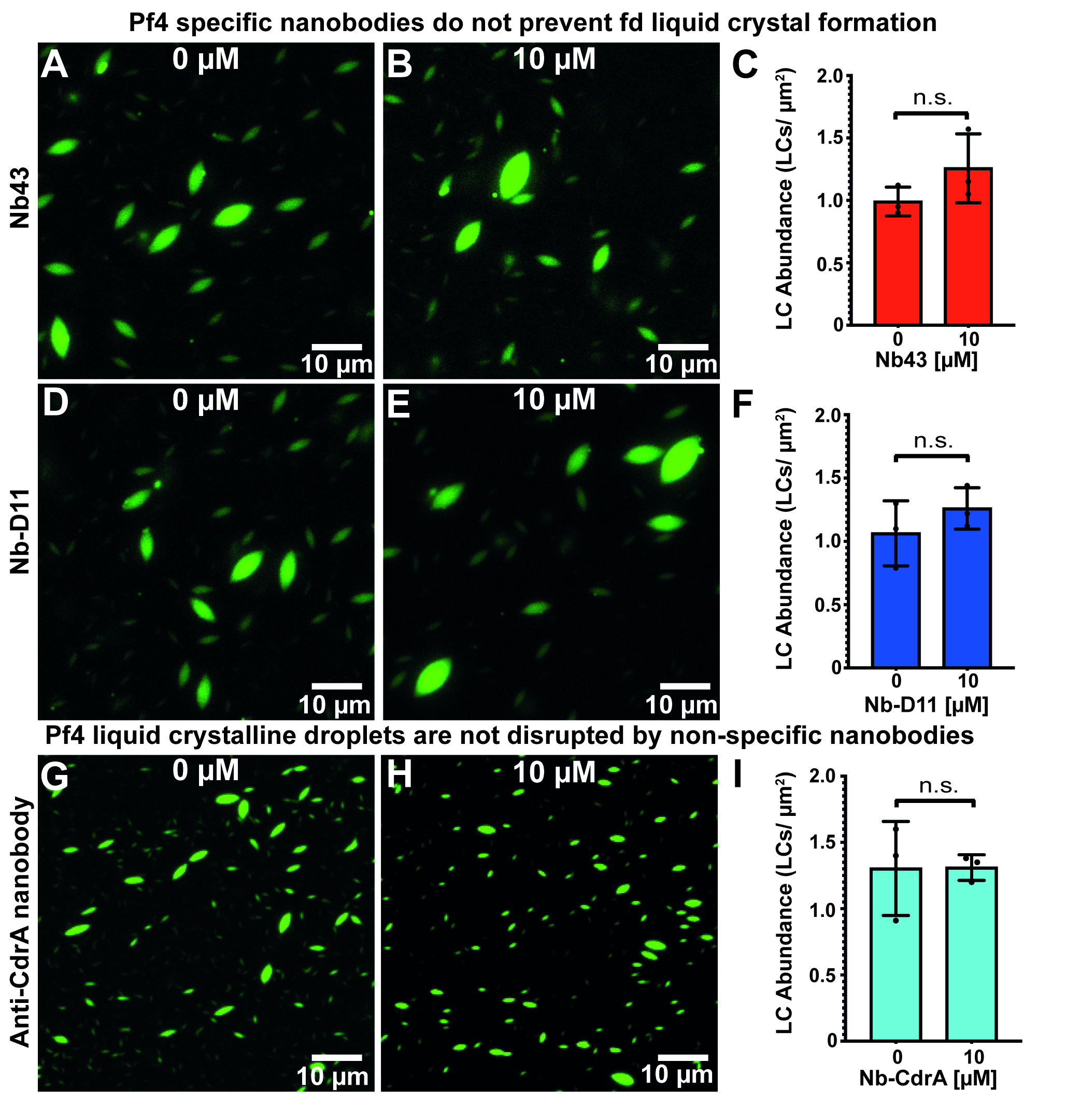

Supplement: S6 Fig — (A and B) Representative light microscopy images of A488-labeled fd liquid crystals formed in the presence of (A) no Nb43 or (B) 10 μM Nb43. (C) Bar chart showing the abundance of fd liquid crystals per μm2. Addition of 10 μM Nb43 did not result in a statistically significant change in liquid crystal formation. (D and E) Representative light microscopy images of A488-labeled fd liquid crystals formed in the presence of (D) no Nb-D11 or (E) 10 μM Nb-D11. (F) Bar chart showing the abundance of fd liquid crystals per μm2. The addition of 10 μM Nb-D11 did not result in a statistically significant change in liquid crystal formation. (G and H) Representative light microscopy images of A488-labeled Pf4 liquid crystalline droplets formed in the presence of (G) no nanobody or (H) 10 μM anti-CdrA nanobody. (I) Bar chart showing the abundance of Pf4 liquid crystalline droplets per μm2. The addition of 10 μM anti-CdrA nanobody did not result in a statistically significant change in liquid crystalline droplet formation. All values are representative of 30 images from three independent replicates. Error bars represent standard deviation. P-values were calculated using an unpaired t test. All images have been background subtracted. The data underlying this Figure can be found in S1 Data. (TIF) [file pbio.3003834.s006.tif]

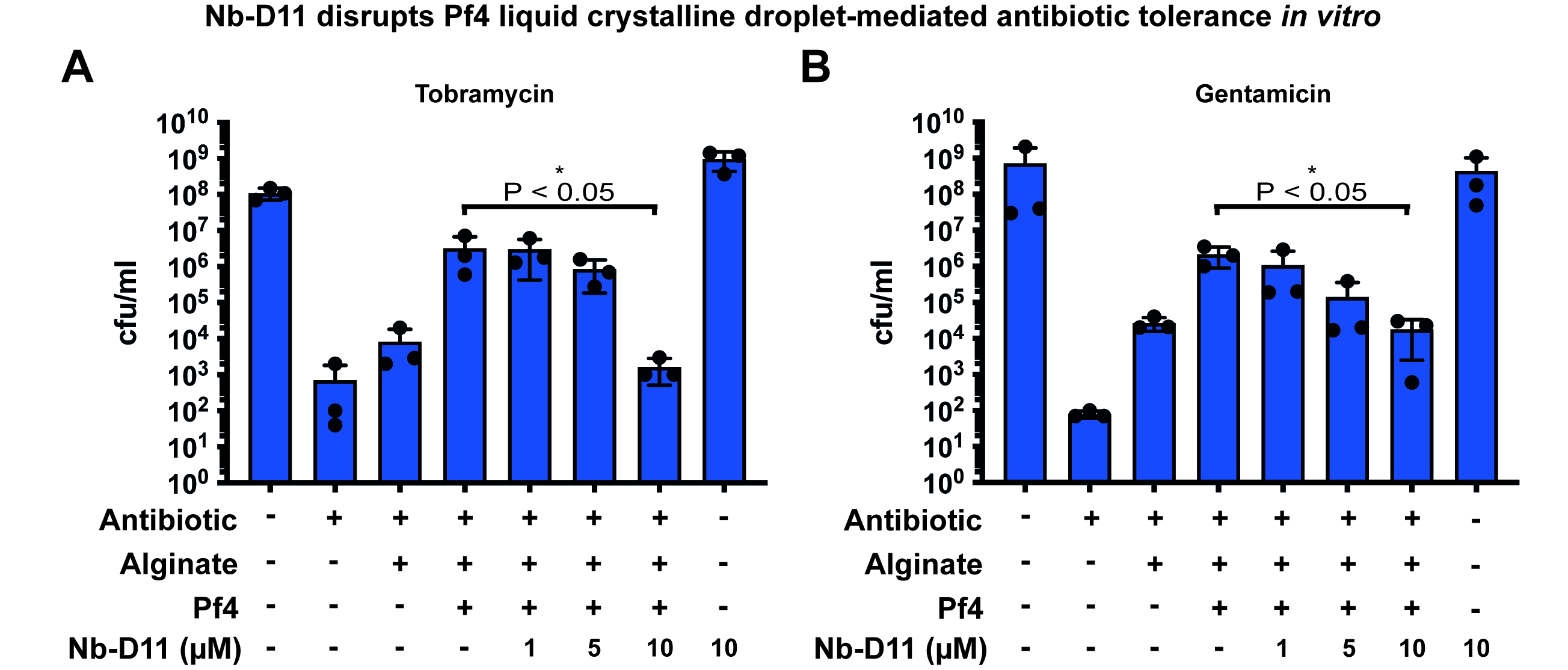

Supplement: S9 Fig — (A and B) Bar graph shows colony-forming units (cfu) per ml (y axis), a measure of P. aeruginosa culture cell viability after (A) tobramycin and (B) gentamicin treatment in the presence of different reagents (x axis). For both antibiotics, 10 µM Nb-D11 significantly reduced antibiotic tolerance levels to that seen in the alginate alone condition (Pvalue < 0.05). Values shown are the mean of three independent experiments. Error bars represent standard deviation. P-values were calculated using an unpaired t test. The data underlying this Figure can be found in S1 Data. (TIF) [file pbio.3003834.s009.tif]

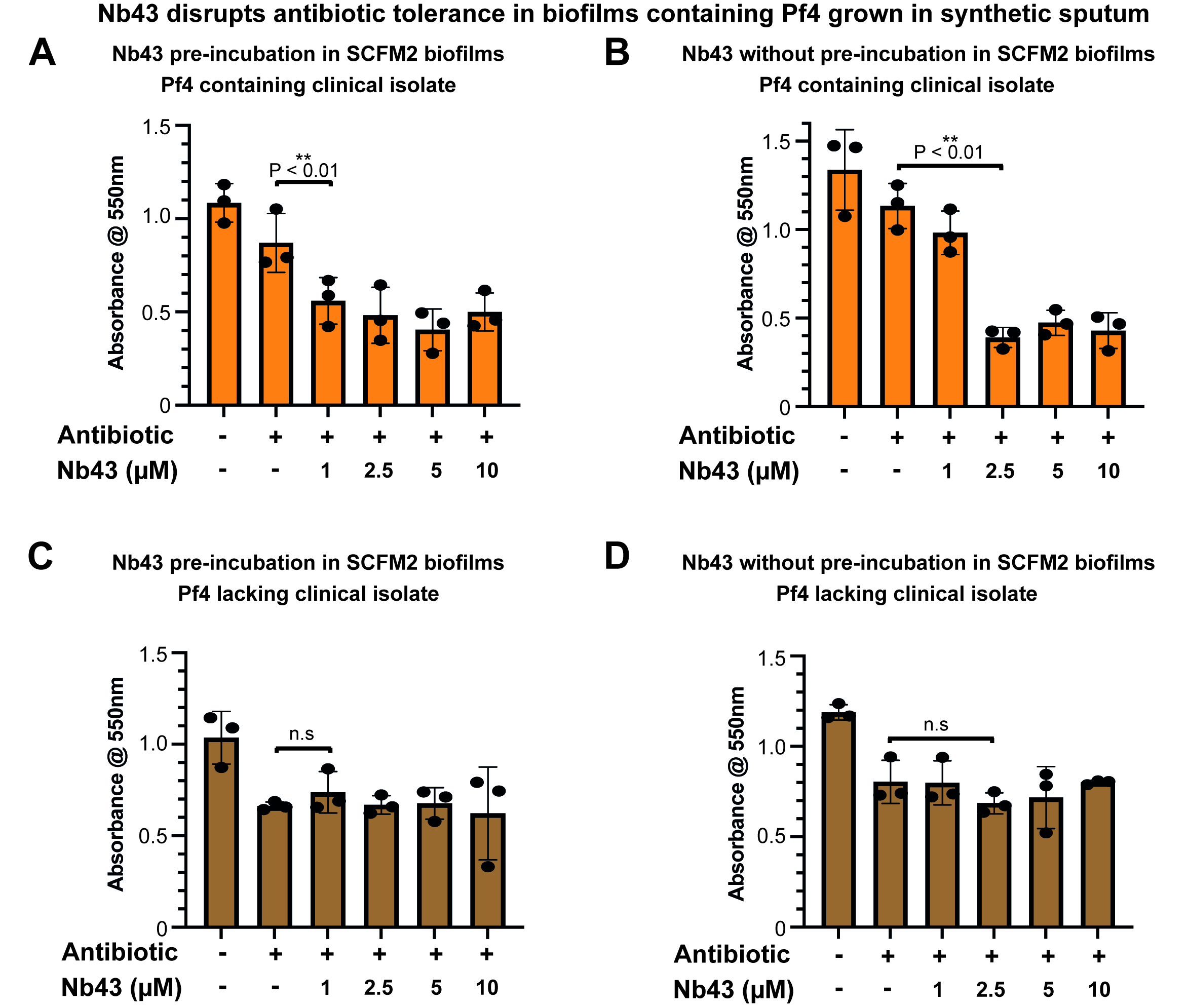

Supplement: S10 Fig — (A and B) A P. aeruginosa clinical strain encoding Pf4 major coat protein, CoaB, was used to grow static biofilms in 96-well plates in SCFM2 media and treated with Nb43 either (A) at the inoculation stage (pre-incubation) or (B) after 24 hours growth (without pre-incubation) with the indicated concentrations of Nb43. At the 24 hours time point, 1 μg/ml tobramycin was added and cultures incubated for a further 8 hours, before plates were treated with crystal violet to assay for biofilm growth. Significantly less biofilm is present with (A) 1 μM Nb43 in pre-incubation conditions (Pvalue < 0.01) and (B) 2.5 μM Nb43 in the without pre-incubation condition (Pvalue < 0.01) compared to the control without Nb43. (C and D) A P. aeruginosa clinical strain lacking Pf4 major coat protein, CoaB, was used to grow static biofilms in 96-well plates in SCFM2 media and treated with Nb43 either (C) at the inoculation stage (pre-incubation) or (D) after 24 hours growth (without pre-incubation) with the indicated concentrations of Nb43. At the 24 hours time point, 1 μg/ml tobramycin was added and cultures incubated for a further 8 hours, before plates were treated with crystal violet to assay for biofilm growth. Graphs show absorbance at 550 nm (crystal violet signal indicating amount of biofilm) on the y axis and components added to the assay on the x axis. Addition of Nb43 had no effect on biofilms formed with the clinical strain lacking CoaB in both (C) pre-incubation and (D) without pre-incubation conditions as compared to the control without Nb43. In all cases, mean values from three replicates are plotted. Error bars represent standard deviation. P-values were calculated using an unpaired t test. The data underlying this Figure can be found in S1 Data. (TIF) [file pbio.3003834.s010.tif]
