# Supplementary material for: Disrupting phage liquid crystalline droplets restores antibiotic susceptibility in Pseudomonas aeruginosa biofilms
Source: PLoS Biol. 2026 Jun 5;24(6):e3003834. doi: 10.1371/journal.pbio.3003834 (PMC13262939; doi:10.1371/journal.pbio.3003834)
Supplement: S1 Raw Images — Original, uncropped images supporting all gels. (PDF) [file pbio.3003834.s012.pdf]

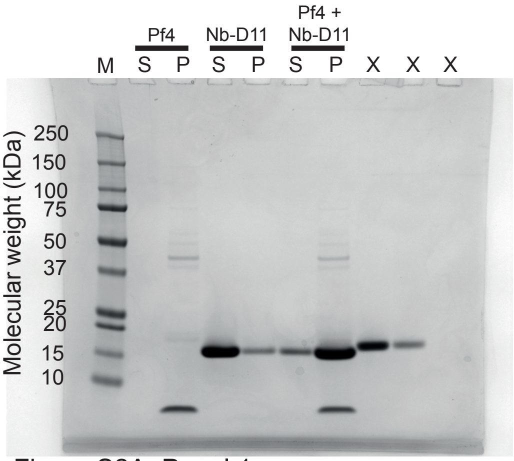

Figure S2A, Panel 1

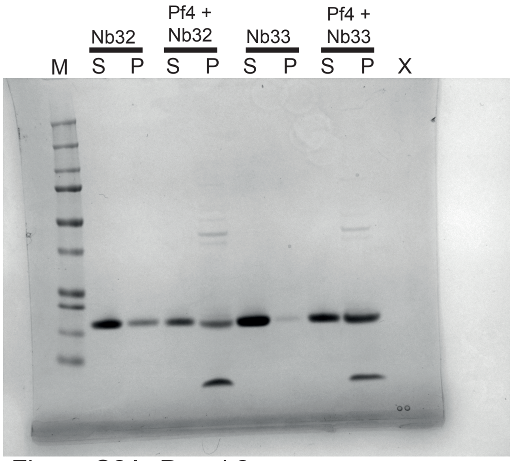

Figure S2A, Panel 2

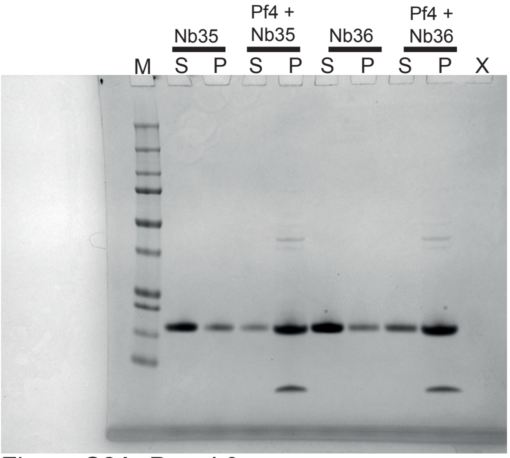

Figure S2A, Panel 3

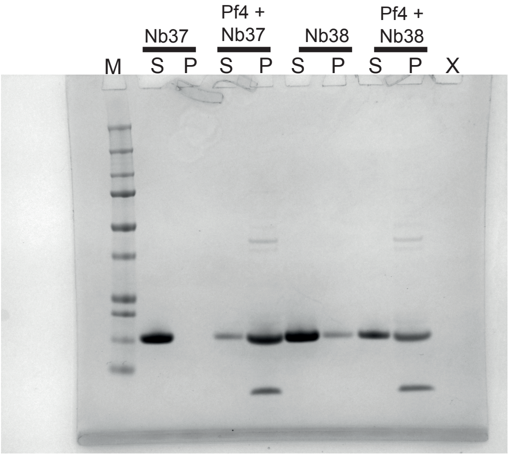

Figure S2A, Panel 4

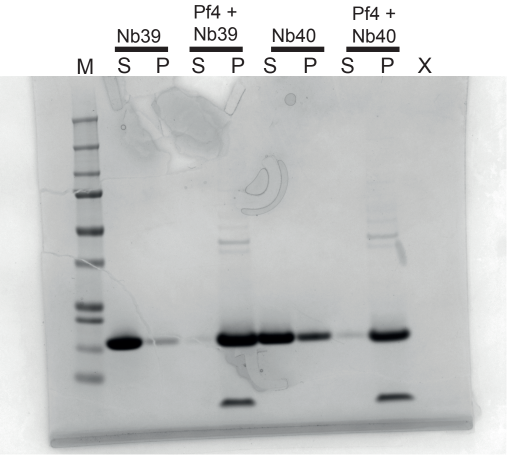

Figure S2A, Panel 5

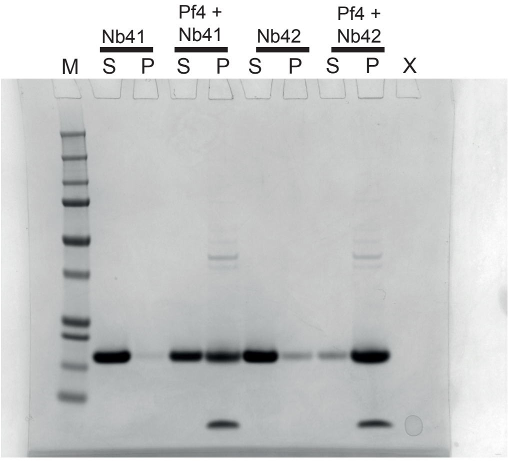

Figure S2A, Panel 6

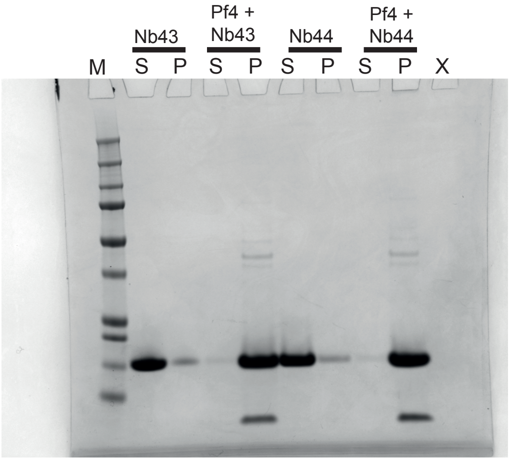

Figure 1C and Figure S2A, Panel 7

M = All blue protein marker (Bio-Rad, cat no. 161-0373)
S = Supernatant
P = Pellet
X = Lane not used in figure

Gels imaged with Bio-Rad XR+ Gel-Documentation system with Coomassie blue protocol

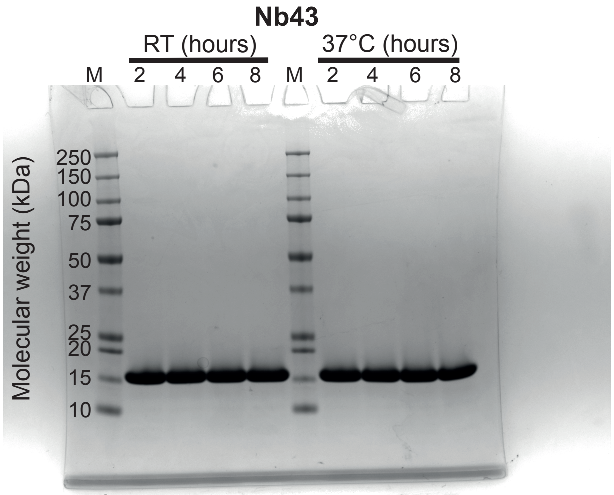

Figure S3B

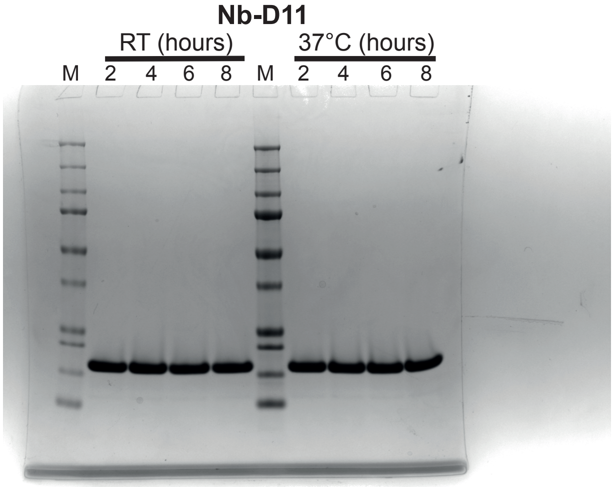

Figure S3D

M = All blue protein marker (Bio-Rad, cat no. 161-0373)
Gels imaged with Bio-Rad XR+ Gel-Documentation system with Coomassie blue protocol
