# Supplementary material for: Disrupting phage liquid crystalline droplets restores antibiotic susceptibility in Pseudomonas aeruginosa biofilms
Source: PLoS Biol. 2026 Jun 5;24(6):e3003834. doi: 10.1371/journal.pbio.3003834 (PMC13262939; doi:10.1371/journal.pbio.3003834)
Supplement: Attachment — Submitted filename: 20260114_Tarafder_et_al_revision_plan.pdf [file pbio.3003834.s015.pdf]

Revision Plan

Review COMMONS

Manuscript number: RC-2025-03209
Corresponding author(s): Abul, Tarafder and Tanmay, Bharat

[The "revision plan" should delineate the revisions that authors intend to carry out in response to the points raised by the referees. It also provides the authors with the opportunity to explain their view of the paper and of the referee reports.

The document is important for the editors of affiliate journals when they make a first decision on the transferred manuscript. It will also be useful to readers of the reprint and help them to obtain a balanced view of the paper.

If you wish to submit a full revision, please use our "Full Revision" template. ***It is important to use the appropriate template to clearly inform the editors of your intentions.***

1. General Statements [optional]

This section is optional. Insert here any general statements you wish to make about the goal of the study or about the reviews.

We thank the Review Commons editors and reviewers for their careful evaluation of the manuscript and their positive comments on the work presented. We are pleased that all reviewers commented on the significance of this work to both microbiologists and clinicians, and its contribution to tackling the major clinical burden of biofilms and antibiotic tolerance. We provide a point-by-point response to the reviewer comments below.

2. Description of the planned revisions

Insert here a point-by-point reply that explains what revisions, additional experimentations and analyses are planned to address the points raised by the referees.

Reviewer #1 (authors' response in blue):
Evidence, reproducibility and clarity
The manuscript by Tarafder et al. describes an interdisciplinary approach, combining biophysical modeling and microbiology, to target antibiotic tolerance in P. aeruginosa biofilms. A key conceptual contribution is the strategy of inhibiting a biophysical mechanism instead of a biochemical interaction. The study is logically organized, advancing from a theoretical model to the design of effective nanobody inhibitors, which are then validated across a series of experimental systems, from in vitro assays to complex static and flow-cell biofilms. The data robustly support the authors' conclusions, suggesting a potentially valuable approach for managing biofilm-based infection. Overall, this is a very interesting and robust study. The

conclusions are well-supported by the evidence provided, and the manuscript is well-written, with figures that effectively illustrate the key results.

We thank the reviewer for this positive summary of the manuscript.

Major comments:
1. The fundamental characteristics of Nb43 and Nb-D11 (e.g., affinity, stability) should be provided…

We have already performed affinity measurements by surface plasmon resonance (SPR), which will be included in the supplement of the revised manuscript. Briefly, Nb43 has approximately 10-fold higher affinity (69 ± 16 nM) for Pf4 than Nb-D11 (650 ± 60 nM).

We will also provide a gel in the supplement showing stability of the nanobodies over time at ambient conditions, as well as at 37 °C.

To solidify the central claim, the direct interaction between CoaB and Nb43 should be confirmed using an orthogonal biochemical method. urthermore, it is important to test whether Nb43 binds to the CoaB proteins from Pf1/Pf5/Pf6 to assess its specificity and broad application in other PA hosts such as MPAO1 and PA14.

We are sorry this was not clear. We will provide a new supplementary difference map between the Pf4 density[1] and the map of Pf4+Nb43. Our results will show that Nb43 is directly interacting with CoaB. We will also add the relevant text to support our revised figures.

Pf1, Pf4 and Pf6 phages have the same CoaB amino acid sequence, meaning that Nb43 would very likely bind to these phages in the same way[2]. We will highlight this point in the text of the revised manuscript.

Pf5 phage, which is expressed in strain PA14, shows high sequence identity with Pf4 when their CoaB proteins are compared (87%)[2]. We will highlight this point in a sequence alignment in the revised manuscript. Furthermore, in Figure 5 we have showed that Nb43 makes both PAO1 biofilms (Figure 5a-b) as well as PA14 biofilms (Figure 5c-k) more susceptible to antibiotics, suggesting that Nb43 is active against both Pf4 (in PAO1) and Pf5 (in PA14). We will discuss the broad application and limitations of Nb43 in respect to phage binding further in the discussion.

2. In the static biofilm assay (Fig. 5a-b), the use of crystal violet staining only reports total biomass. To clarify the mechanism of action, experiments should distinguish whether Nb43 primarily prevents biofilm attachment/formation or actively eradicates an established biofilm. This is particularly relevant for the pre-incubation condition.

We completely agree, which is why we added the nanobody against mature biofilms (i.e. after biofilm formation) at a late time point in Figure 5b as well as before biofilms were formed (Figure 5a). We are sorry if this was unclear. We will amend the revised manuscript with a small schematic in the supplement, making this important point clear.

3. The discussion should address the limitations of this therapeutic approach. A key concern is the potential for Pf4 reinfection and subsequent relapse of chronic infection, which is a major challenge in the field. Additionally, the manuscript would be strengthened by a more critical and direct comparison of this Nb-based strategy against existing anti-virulence or anti-biofilm alternatives, highlighting its potential advantages and drawbacks.

We will add a paragraph to the discussion to address these topics. We agree that contextualizing the Nb-based strategy, and its limitations and advantages in comparison to other existing treatments, is important.

Minor comments

1. The prevention of Pf activation in P. aeruginosa biofilms is an important aspect that should be addressed in the Introduction and Discussion.

We will amend the text to cover Pf activation by genetic (Pf4 repressors, superinfection repression) and environmental factors (reducing oxidative stress, quorum sensing) in the introduction and discussion.

2. In the Methods section for the biophysical model, the choice of specific parameters (e.g., phage length a=80 nm, depletant diameter σ=2.4 nm) is justified by referencing the system being modeled. However, a brief sentence explicitly stating that these values were chosen based on the known dimensions of Pf4 and alginate would be helpful for readers that are not familiar with the system.

We will add a sentence to the methods section to explain why these numbers were chosen.

Significance

This study provides a mechanistic insight into the advance and offers a complementary approach to treating biofilm-related infections, which remains an unexplored area in the field. The reported findings are likely to be of interest and significance to microbiologists and clinicians concerned with biofilm infections.

Thank you.

My own expertise lies in the genetic and biochemical aspects of prophage induction and biofilm formation. Therefore, the details of nanobodies and their potential side effects fall outside the scope of my evaluation.

Reviewer #2 (authors' response in blue):

Revision Plan

Evidence, reproducibility and clarity

In the well-written manuscript by Tarafder et al., the authors follow up on their previous investigations of the filamentous bacteriophage Pf4, which self-assembles into a crystalline droplet surrounding Pseudomonas aeruginosa cells within a biofilm. Using theoretical coarse-grained molecular dynamics (MD) simulations, they predict that binding a small molecule or protein to the surface of bacteriophage Pf4 should disrupt the attraction-in this case depletion attraction-between individual phage particles. To test this hypothesis, nanobodies were raised against Pf4, and two promising candidates, Nb43 and Nb-D11, were identified. These nanobodies were characterized using biochemical assays, and binding of Nb43 to CoaB, the major coat protein, was visualized using cryo-EM. Using fluorescence microscopy and cryo-ET, the authors convincingly demonstrate that nanobodies can disrupt Pf4 crystalline droplet formation. Strikingly, nanobody-mediated disruption of Pf4 droplets also increases antibiotic susceptibility of P. aeruginosa both in vitro and in biofilm settings.

We thank the reviewer for this positive summary of the manuscript.

Major comments

1) Theoretical modelling:

The MD simulations, as currently presented, do not add conceptual depth to the study. The idea that blocking an interaction site between phages (whether through active-site interference, obstruction of a protein-protein interface, or simple steric hindrance) would prevent alignment is straightforward and does not necessarily require MD simulations to justify. As such, this section feels superfluous and is currently the weakest point in an otherwise strong manuscript. Unless the simulations can meaningfully address at least some of the questions listed below, the authors should consider removing this part:

The MD simulation is very simplistic, and filamentous phages are clearly not hard rods, as seen in the cryo-EM images. Would a certain degree of Pf4 flexibility allow to stabilize droplets even in the presence of low concentrations of Pf4 binders?

How do the MD simulations explain that already pre-formed crystalline droplets can be penetrated and disassembled by small Pf4 binders?

The authors state that Pf4 binders must be large relative to the depletant particles. Can this be demonstrated experimentally? Is there a sweet spot, as large molecules potentially cannot penetrate preformed droplets?

It is true that, given what we could show experimentally in the rest of the manuscript, the modelling does not alter or change our conclusions concerning successful nanobody treatments of biofilms. However, when performing this study, the modelling was an important first step, which enabled us to commence the experimental work in the first place. In particular, the modelling allowed us to identify and validate a potential biophysical mechanism by which nanobodies block the depletion interaction between phages (we note that there is no classical "protein:protein" interface between phages, but rather a lateral association governed by liquid crystalline parameters of the droplet, which is why blocking the interaction was non-trivial). We also agree with the Reviewer's

comments that we should address modelling assumptions, and we will therefore clearly highlight model limitations in the text of the revised manuscript.

We are keen to take into consideration the reviewer's criticism, therefore we propose to combine the key finding in the current Figure 1 regarding small binder density (Figure 1e-f) with current Figure 2 to generate a new revised Figure 1. The rest of the information in current Figure 1 would be presented in a new Extended Data Figure 1. We believe this change will balance the emphasis of the manuscript and address reviewer 2's comments.

2) Nanobody penetration into crystalline droplets (Extended Data Fig. 6a-d) vs. antibiotic penetration (Fig. 4)
The authors show that Nb43 penetrates Pf4 droplets even at concentrations that do not disrupt droplet stability. How do the authors explain that a relatively large nanobody penetrates the crystalline droplet, whereas a much smaller antibiotic does not diffuse trough the droplet?

This is an intriguing question that we cannot fully answer at this point. Our best hypothesis is that the high-affinity binding of Nb43 to Pf4 promotes its retention and association with the droplets, compared to the antibiotic, which does not bind to the droplets (even when the droplet is made of phages with or without DNA[1]). We will discuss this point in the revised manuscript text.

In the experiments shown in Figure 4, the authors assess antibiotic activity against P. aeruginosa in the presence of Pf4 crystalline droplets. If I understand correctly, the additionally added Pf4 droplets do not physically encompass the bacteria, yet they still reduce antibiotic tolerance. If so, this appears to contradict the conclusion that Pf4 droplets act primarily as a diffusion barrier (as stated in the section title). Instead, this would suggest that Pf4 may reduce antibiotic potency through another mechanism (e.g., direct binding or sequestration).

Our apologies for not being clearer here. The added Pf4 liquid crystalline droplets encapsulate cells in the assay presented in Figure 4. We will add a schematic in the revised manuscript to make this clear for the readers.

We will also discuss alternative mechanisms in the discussion, which may partly explain our observations, and which could be the subject of future mechanistic studies.

Would it be possible to test the addition of Pf4 alone, without the biopolymer alginate, to determine whether Pf4 itself is sufficient to increase antibiotic tolerance?

We will repeat the assays in Figure 4a-b in this manuscript including a Pf4 alone control in the revised manuscript.

Minor comments:
- Title: The title is overstated. Please consider changing it to something similar to:
"Targeted disruption of phage liquid crystalline droplets abolishes antibiotic tolerance in

Pseudomonas aeruginosa biofilms."

We will change the title as suggested.

- Introduction sentence:
"...where filamentous phage particles align along their axis in the presence of biopolymer,..."
Please introduce what biopolymers are and specify which types are relevant here.

We will define biopolymers in the introduction.

- Amorphous Pf4 aggregates after Nb43 treatment (Fig 3b,e):
The authors should discuss the nature of these aggregates. It appears that smaller spindles are both broken up and impeded in their formation after Nb43 treatment, whereas larger aggregates seem to persist.

We agree with the reviewer's observation. We will add some sentences in the results section to highlight this point.

- Fig. 3c and 3f:
Please describe how liquid crystalline structures were defined in the fluorescence images. Were thresholds for size, intensity, or morphology applied?

We will provide this information in the methods section.

- Use of *P. aeruginosa* ΔPAO728:
For clarity, please explain why the strain lacking the Pf4 integrase is included in the in-vitro assays.

We will add a sentence to the results explaining this. Briefly, *P. aeruginosa* ΔPAO728, lacking the Pf4 integrase, does not produce Pf4 phage ensuring the only Pf4 phage present is the purified phage added in the assay.

-Discussion:
Neisseria meningitidis and Vibrio cholerae use filamentous phages to increase virulence. Do these phages also form liquid crystalline droplets? If not, how do the authors envision that the nanobody strategy described here could be applied to prevent infection? In general, the findings are hard to generalize to other biofilms matrices, which are highly heterogenous.

This is a fair point. All rod-shaped inoviruses will have the propensity to form liquid crystalline structures under the appropriate depletant conditions, but so far they have not been studied in *Neisseria meningitidis* and *Vibrio cholerae* biofilms. Nevertheless, we believe that the strategy adopted to disrupt *P. aeruginosa* Pf aggregates in this manuscript could be applicable to other non-phage filamentous molecules that form higher-order structures in the crowded biofilm matrix

environment[3]. Examples of such filaments are *B. subtilis* TasA, *E. coli* Curli or *P. aeruginosa* Fap fibres. We will clarify this point in the discussion section, and give a balanced view on the future perspective of this strategy.

Significance

Bacterial biofilms and their associated antibiotic tolerance represent a major clinical burden, and new strategies to overcome these defenses are urgently needed. The strategy presented here-targeting and disrupting the protective extracellular matrix formed by liquid crystalline Pf4 phage droplets-is an exciting and innovative approach with clear translational potential for combating P. aeruginosa biofilms. The study is experimentally rigorous, well written, and carefully analyzed, and it represents a logical and impactful next step following the group's previous work. This manuscript will have significant impact on the field of P. aeruginosa biofilm research by providing a mechanistically grounded method to disrupt the protective biofilm architecture. However, it is important to note that the extracellular matrix architecture of biofilms formed by other bacterial species differs substantially, and thus the current findings cannot be directly generalized beyond P. aeruginosa without further investigation.

Thank you.

Description of the revisions that have already been incorporated in the transferred manuscript

Please insert a point-by-point reply describing the revisions that were already carried out and included in the transferred manuscript. If no revisions have been carried out yet, please leave this section empty.

3. Description of analyses that authors prefer not to carry out

Please include a point-by-point response explaining why some of the requested data or additional analyses might not be necessary or cannot be provided within the scope of a revision. This can be due to time or resource limitations or in case of disagreement about the necessity of such additional data given the scope of the study. Please leave empty if not applicable.

References

1 Tarafder, A. K. *et al.* Phage liquid crystalline droplets form occlusive sheaths that encapsulate and protect infectious rod-shaped bacteria. *Proc Natl Acad Sci U S A* **117**, 4724–4731 (2020). https://doi.org/10.1073/pnas.1917726117

2 Fiedoruk, K. *et al.* Two Lineages of Pseudomonas aeruginosa Filamentous Phages: Structural Uniformity over Integration Preferences. *Genome Biol Evol* **12**, 1765–1781 (2020). https://doi.org/10.1093/gbe/evaa146

3 Bohning, J., Tarafder, A. K. & Bharat, T. A. M. The role of filamentous matrix molecules in shaping the architecture and emergent properties of bacterial biofilms. *Biochem J* **481**, 245–263 (2024). https://doi.org/10.1042/BCJ20210301
