# Supplementary material for: Disrupting phage liquid crystalline droplets restores antibiotic susceptibility in Pseudomonas aeruginosa biofilms
Source: PLoS Biol. 2026 Jun 5;24(6):e3003834. doi: 10.1371/journal.pbio.3003834 (PMC13262939; doi:10.1371/journal.pbio.3003834)
Supplement: Attachment — Submitted filename: Response_Tarafder_etal_final.pdf [file pbio.3003834.s017.pdf]

__Response to reviewers__

Manuscript number: PBIOLOGY-D-26-00139 (RC-2025-03209)
Corresponding author(s): Abul, Tarafder and Tanmay, Bharat

We thank the PLOS Biology editors and reviewers for their careful evaluation of the manuscript and their positive comments on the work presented. We are pleased that all reviewers commented on the significance of this work to both microbiologists and clinicians, and its contribution to tackling the major clinical burden of biofilms and antibiotic tolerance. We provide a point-by-point response to the academic editor and reviewer comments below.

Academic editor request (authors' response in blue):

Show dependency of effect on pf4 genomic presence/absence. Currently much (all?) of the work features pf4 that is exogenously supplied, in simple media. It would increase confidence in both the causal claim and the real-world relevance of this result to show that the effect is present in a pf4+ strain of P aeruginosa, and can be ablated by genetic deletion of pf4. Showing this in a more relevant media (eg synthetic sputum media) would further buttress the real-world relevance.

We thank the academic editor for their suggestions and agree this would increase the real-world relevance of the manuscript. To address these points, we have performed new experiments where we demonstrate that Nb43 can disrupt Pf4 liquid crystalline droplets in the synthetic cystic fibrosis sputum media SCFM2 (Figure S7J-L, see adjoining).

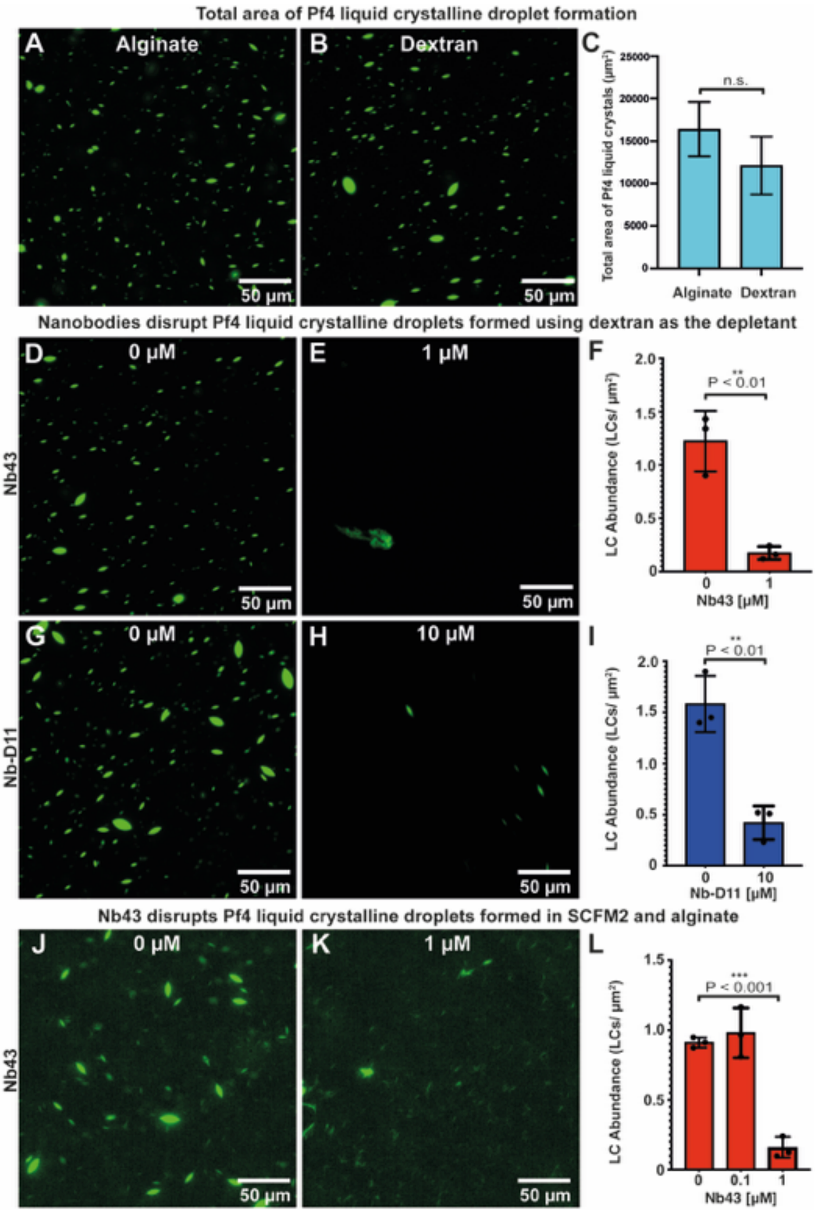

Figure S7. Nanobodies disrupt Pf4 liquid crystalline droplet formation with different media and depletants. (**A-B**) Representative light microscopy images with comparable total liquid crystalline droplet area formed by A488- labelled Pf4 using (A) alginate and (B) dextran as the depletant. **(C)** Bar chart showing total liquid crystalline droplet area as assessed by light microscopy followed by image segmentation of droplets. (**D-E**) Representative light microscopy images of A488-labelled Pf4 liquid crystalline droplets formed with dextran as the depletant in the presence of (D) no Nb43 or (E) 1 μM Nb43. (**F**) Bar chart showing the abundance of Pf4 liquid crystalline droplets per μm^2. Addition of 1 μM Nb43 results in a statistically significant reduction in liquid crystalline droplet formation (P_{value} < 0.01). (**G-H**) Representative light microscopy images of A488-labelled Pf4 liquid crystalline droplets formed with dextran as the depletant in the presence of (G) no Nb-D11 or (H) 10 μM Nb-D11. (**I**) Bar chart showing the abundance of Pf4 liquid crystalline droplets per μm^2. Addition of 10 μM Nb-D11 results in a statistically significant reduction in liquid crystalline droplet formation (P_{value} < 0.01). (**J-L**) Representative light microscopy images of A488-labelled Pf4 liquid crystalline droplets formed in synthetic sputum medium SCFM2 with alginate as the depletant in the presence of (J) no Nb43 or (K) 1 μM Nb43. (**I**) Bar chart showing the abundance of Pf4 liquid crystalline droplets per μm^2. Addition of 1 μM Nb43 results in a statistically significant reduction in liquid crystalline droplet formation (P_{value} < 0.001).

We have also added the following accompanying text.

Results, line 205:
Finally, we tested the ability of Nb43 to disrupt liquid crystalline droplets in the presence of synthetic cystic fibrosis sputum media (SCFM2), a medium containing mucin, DNA and lipids providing a nutritional and physical environment similar to sputum (51). Nb43 at 1 µM concentration could effectively disrupt Pf4 liquid crystalline droplets in this physiologically relevant medium, the same concentration as in other media (Fig S7J-L)

We have also performed new static biofilm disruption assays using *P. aeruginosa* clinical isolates grown in SCFM2. Here we tested isolates where the Pf4 major coat protein, CoaB, was present or absent in the bacterial genome. Briefly, we find that Nb43 increases antibiotic susceptibility of the bacteria containing Pf4, but has no effect on antibiotic susceptibility of the clinical isolate bacteria not encoding Pf4. This data is presented in a new figure (Fig S10A-D).

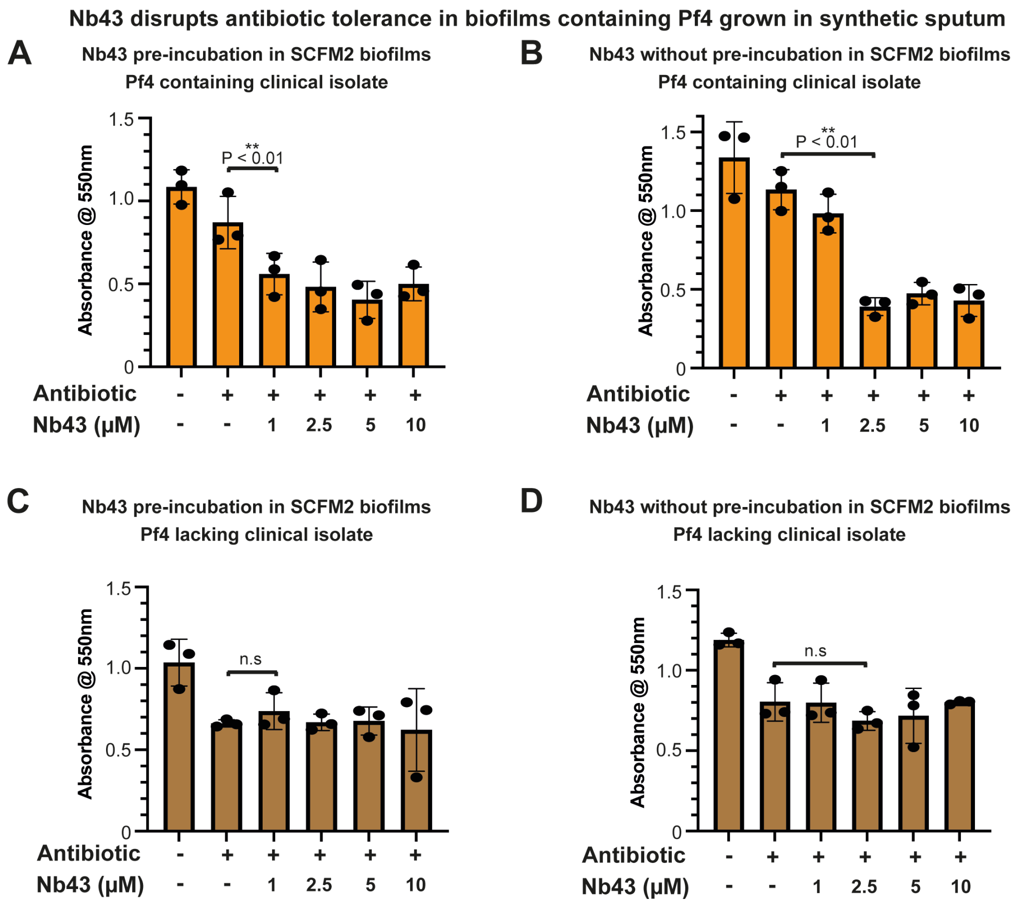

Figure S10. Nb43 action on biofilms of clinical isolates grown in synthetic sputum media. (A-B) A *P. aeruginosa* clinical strain encoding Pf4 major coat protein, CoaB, was used to grow static biofilms in 96-well plates in SCFM2 media and treated with Nb43 either (A) at the inoculation stage (pre-incubation) or (B) after 24 hours growth (without pre-incubation) with the indicated concentrations of Nb43. At the 24 hours timepoint, 1 µg/ml tobramycin was added and cultures incubated for a further 8 hours, before plates were treated with crystal violet to assay for biofilm growth. Significantly less biofilm is present with (A) 1 µM Nb43 in pre-incubation conditions (P_{value} < 0.01) and (B) 2.5 µM Nb43 in the without pre-incubation condition (P_{value} < 0.01) compared to the control without Nb43. (**C-D**) A *P. aeruginosa* clinical strain lacking Pf4 major coat protein, CoaB, was used to grow static biofilms in 96-well plates in SCFM2 media and treated with Nb43 either (C) at the inoculation stage (pre-incubation) or (D) after 24 hours growth (without pre-incubation) with the indicated concentrations of Nb43. At the 24 hours timepoint, 1 µg/ml tobramycin was added and cultures incubated for a further 8 hours, before plates were treated with crystal violet to assay for biofilm growth. Graphs show absorbance at 550 nm (crystal violet signal indicating amount of biofilm) on the y axis and components added to the assay on the x axis. Addition of Nb43 had no effect on biofilms formed with the clinical strain lacking CoaB in both (C) preincubation and (D) without pre-incubation conditions as compared to the control without Nb43. In all cases, mean values from three replicates are plotted. Error bars represent standard deviation. *P*-values were calculated using an unpaired *t*-test.

We also added the following accompanying text.

Results, line 270:

Next, we tested the ability of Nb43 to disrupt biofilms grown in the physiologically relevant SCFM2 medium, with genomically characterised clinical isolates (52) where the Pf4 coat protein, CoaB, was present or absent in the genome. Under pre-incubation conditions, 1 μM Nb43 significantly reduced biofilm growth for the Pf4 containing clinical strain grown in SCFM2 ($P_{value} < 0.01$) and without pre-incubation a higher concentration of 2.5 μM was required ($P_{value} < 0.01$) (Fig S10A-B), mirroring the results obtained for PAO1 grown in LB medium (Fig 4A-B). Nb43 had no effect on the biofilm growth of the clinical isolate that lacked Pf4 at any concentration. Interestingly, the biofilms from the Pf4 lacking strain did show some tolerance to antibiotic, suggesting that another Pf4-independent mechanism of tolerance may be operating (Fig S10).

Reviewer #1 (authors' response in blue):

Evidence, reproducibility and clarity

The manuscript by Tarafder et al. describes an interdisciplinary approach, combining biophysical modeling and microbiology, to target antibiotic tolerance in P. aeruginosa biofilms. A key conceptual contribution is the strategy of inhibiting a biophysical mechanism instead of a biochemical interaction. The study is logically organized, advancing from a theoretical model to the design of effective nanobody inhibitors, which are then validated across a series of experimental systems, from in vitro assays to complex static and flow-cell biofilms. The data robustly support the authors' conclusions, suggesting a potentially valuable approach for managing biofilm-based infection. Overall, this is a very interesting and robust study. The conclusions are well-supported by the evidence provided, and the manuscript is well-written, with figures that effectively illustrate the key results.

We thank the reviewer for this positive summary of the manuscript.

Major comments:

1. The fundamental characteristics of Nb43 and Nb-D11 (e.g., affinity, stability) should be provided…

We have performed affinity measurements by surface plasmon resonance (SPR), which are included in the supplement of the revised manuscript. Briefly, Nb43 has approximately 10-fold higher affinity (69 ± 16 nM) for Pf4 than Nb-D11 (650 ± 60 nM) (Fig S2B-E).

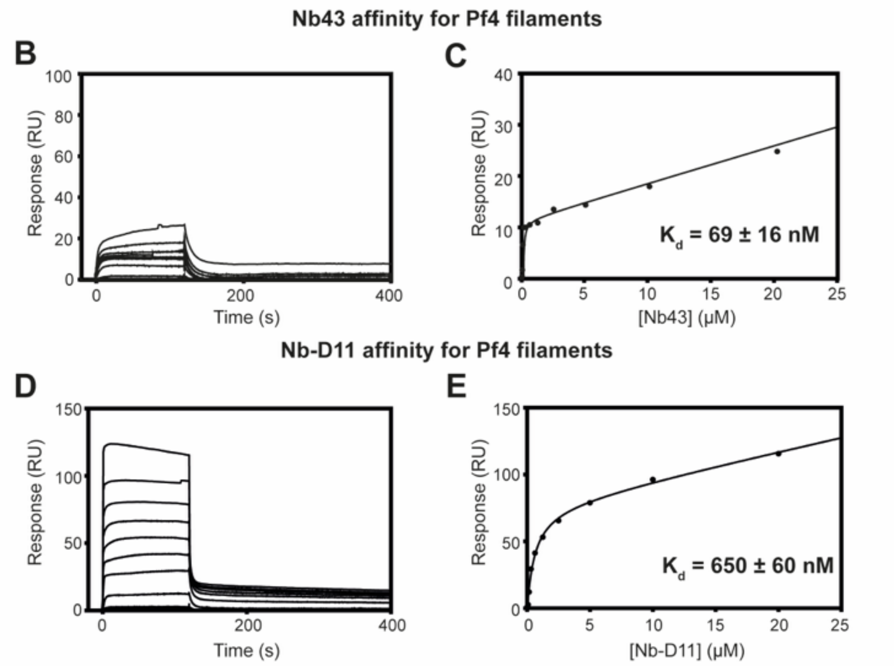

Results, line 148:

Surface plasmon resonance (SPR) analysis confirmed that both Nb43 and Nb-D11 bind to Pf4, but with markedly different affinities (K_d = 69 ± 16 nM for Nb43 and 650 ± 60 nM for Nb-D11, Fig 1D and S2B-E).

We also provide new experiments where we study the stability of the nanobodies by differential scanning fluorimetry (Nb43 Tm = 46.8 °C, Nb-D11 Tm = 44.7 °C) as well as a time-course where we incubated the nanobodies at room temperature or 37 °C and assayed degradation by SDS-PAGE. Both methods indicated that the nanobodies are stable (Fig S3).

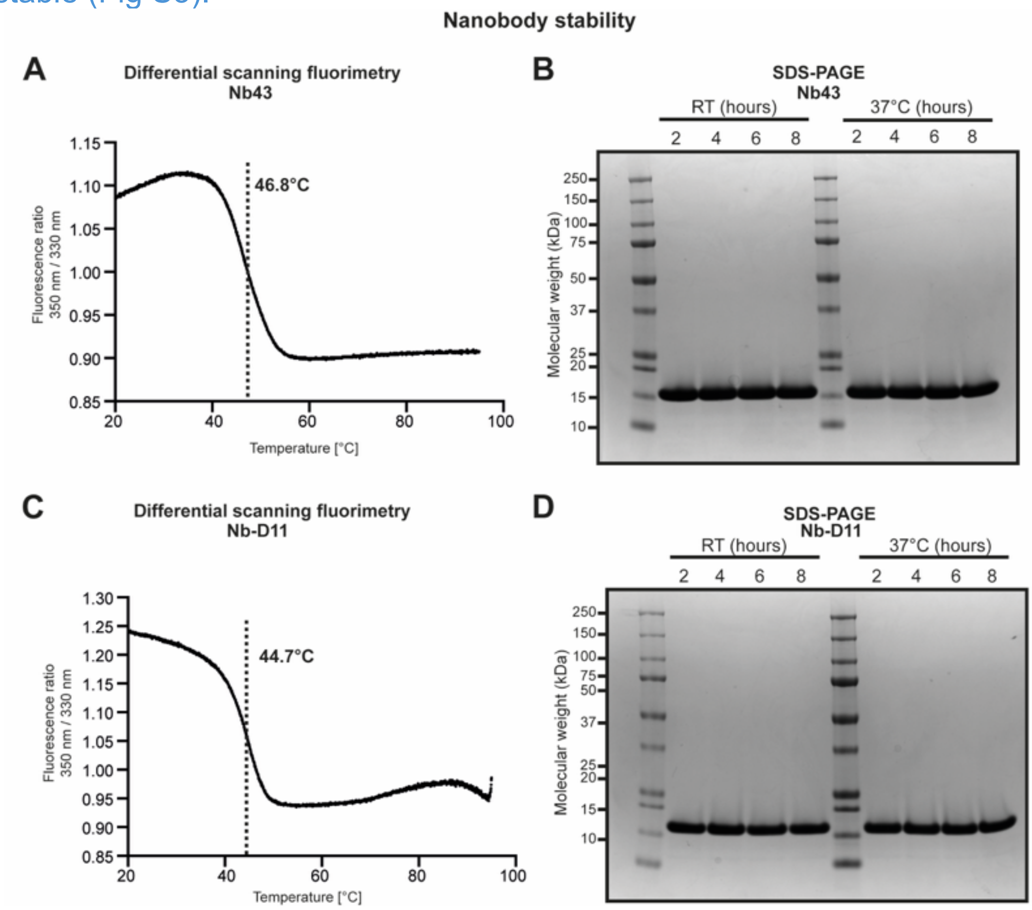

Figure S3. Nanobody stability. (**A**) Differential scanning fluorimetry of Nb43. Melting curve for 1 μM Nb43 over a temperature range of 20-95 °C. Intrinsic fluorescence was measured as the ratio of fluorescence at 350 nm / 330 nm. Nb43 has a Tm of 46.8 °C (**B**) Time-course of Nb43 stability at room temperature (RT) and 37 °C. Nb43 was incubated at the indicated temperature for the indicated time before analysis by SDS-PAGE to assay for protein degradation. (**C**) Differential scanning fluorimetry of Nb-D11. Melting curve for 1 μM Nb-D11 over a temperature range of 20-95 °C. Intrinsic fluorescence was measured as the ratio of fluorescence at 350 nm / 330 nm. Nb-D11 has a Tm of 44.7 °C. (**D**) Time-course of Nb-D11 stability at room temperature (RT) and 37 °C. Nb43 was incubated at the indicated temperature for the indicated time before analysis by SDS-PAGE to assay for protein degradation.

Results, line 150:

Differential scanning fluorimetry analysis indicated that both nanobodies were stable (Tm = 46.8 °C for Nb43 and 44.7 °C for Nb-D11) which was confirmed by SDS-PAGE analysis of nanobody incubated at both room temperature and 37 °C (Fig S3).

To solidify the central claim, the direct interaction between CoaB and Nb43 should be confirmed using an orthogonal biochemical method. urthermore, it is important to test whether Nb43 binds to the CoaB proteins from Pf1/Pf5/Pf6 to assess its specificity and broad application in other PA hosts such as MPAO1 and PA14.

We are sorry this was not clear. We provide a new supplementary difference map between the Pf4 density and the map of Nb43+Pf4 along with a schematic representation of the structural proteins comprising Pf4 (Fig S4). Our results show that Nb43 is directly interacting with CoaB. We have also added the relevant text to support our revised figure.

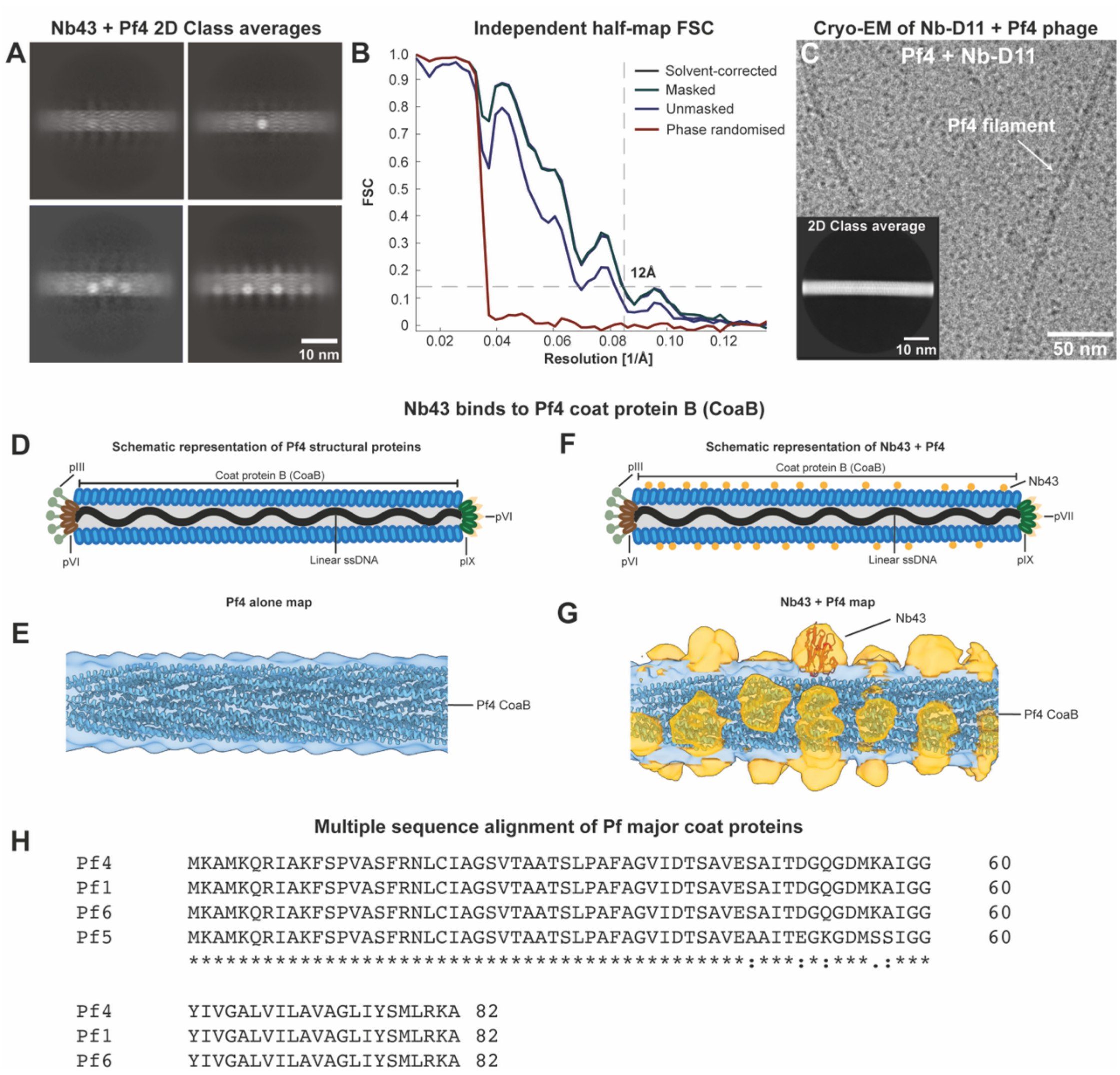

Figure S4. Cryo-EM analysis of Pf4 filaments incubated with nanobodies. (**A**) Gallery of 2D class averages illustrating heterogeneity of Nb43 occupancy on Pf4 filaments. (**B**) Resolution estimation of Pf4-Nb43 reconstruction (Fig 1I) by Fourier shell correlation (FSC) of independently aligned and averaged half-maps. Dashed line indicates 0.143 criterion. (**C**) Cryo-EM micrograph of Pf4 with Nb-D11, showing Pf4 filaments with no apparent decoration. Inset shows a representative 2D class average. (**D**) Schematic representation of Pf4 phage showing distribution of Pf4 structural proteins. (**E**) Cryo-EM density of Pf4 alone (EMDB-10593, low pass filtered to 15 Å) with model fitted in blue. (**F**) Schematic representation of Pf4 phage showing

distribution of Pf4 structural proteins bound to Nb43. (**G**) Map of Pf4-Nb43 with density corresponding to the Pf4 filament coloured blue, and Nb43 density coloured orange. Nb43 density is seen decorating the surface of Pf4 phage showing direct binding to Pf4 CoaB, the sole protein present along the phage filament surface. (**H**) Clustal Omega multiple sequence alignment of CoaB proteins from different Pf phages. * = sequence identity, : = strong sequence homology, . = weak sequence homology.

Results, line 170:
This modeling from our structural data, together with a comparison between the Nb43+Pf4 density with Pf4 without Nb43 (Pf4 alone) density, confirmed that the extra density, not accounted for by the Pf4 phage itself, was positioned outside the phage diameter directly on the surface of the phage, seen along the length of the phage filament. This analysis confirmed that Nb43 binds directly to CoaB, because the sole protein present along the length of the phage is CoaB (Fig 1 I-J and S4D-G).

Pf1, Pf4, Pf5 and Pf6 phages have exactly the same CoaB amino acid sequence at their N-termini, which forms the outermost surface of the phage, meaning that Nb43 would very likely bind to these phages in the same way. We highlight this point in a sequence alignment in the revised manuscript (Fig S4H).

H **Multiple sequence alignment of Pf major coat proteins**

```
Pf4     MKAMKQRIAKFSPVASFRNLCIAGSVTAATSLPAFAGVIDTSAVESAITDGQGDMKAIGG    60
Pf1     MKAMKQRIAKFSPVASFRNLCIAGSVTAATSLPAFAGVIDTSAVESAITDGQGDMKAIGG    60
Pf6     MKAMKQRIAKFSPVASFRNLCIAGSVTAATSLPAFAGVIDTSAVESAITDGQGDMKAIGG    60
Pf5     MKAMKQRIAKFSPVASFRNLCIAGSVTAATSLPAFAGVIDTSAVEAAITEGKGDMSSIGG    60
        *********************************************:***:*:***.:***

Pf4     YIVGALVILAVAGLIYSMLRKA 82
Pf1     YIVGALVILAVAGLIYSMLRKA 82
Pf6     YIVGALVILAVAGLIYSMLRKA 82
Pf5     YIVGALVILAVAGLVYSMLRKA 82
        **************:*******
```

We also added the following accompanying text.

Results, line 161:
The Pf4 CoaB amino acid sequence is conserved in Pf1 and Pf6 phages and shows high sequence identity (87%) to the Pf5 major coat protein (49). Furthermore, the amino acid sequence of all these CoaB proteins is identical at the N-termini, which forms the outer surface of the phage that is recognised by Nb43, suggesting that Nb43 will bind to Pf phages from other *P. aeruginosa* strains in the same manner (Fig S4H).

Furthermore, in Figure 4 we have shown that Nb43 makes both PAO1 biofilms (Figure 4A-B) as well as PA14 biofilms (Figure 4C-K) more susceptible to antibiotics, showing that Nb43 is active against both Pf4 (in PAO1) and Pf5 (in PA14). We discuss the broad application and limitations of Nb43 in respect to phage binding further in the discussion.

Discussion, line 306:
Many strains of *P. aeruginosa* express Pf phages with highly similar major coat protein, with their N-terminal sequences identical to Pf4, such as Pf6 in MPAO1 and Pf5 in PA14, hence nanobodies targeting Pf phages could have multiple use cases against different strains of *P. aeruginosa.* However, nanobodies presented in this study are

limited to *P. aeruginosa* strains harbouring phages expressing similar major coat protein to Pf4.

2. In the static biofilm assay (Fig. 5a-b), the use of crystal violet staining only reports total biomass. To clarify the mechanism of action, experiments should distinguish whether Nb43 primarily prevents biofilm attachment/formation or actively eradicates an established biofilm. This is particularly relevant for the pre-incubation condition

We completely agree, which is why we added the nanobody against mature biofilms (i.e. after biofilm formation) at a late time point in Figure 4B to compare with a case where the nanobody was applied before biofilms were formed (Figure 4A). We are sorry if this was unclear. We have amended the revised manuscript with a small schematic in the figure, making this important point clear (Fig 4).

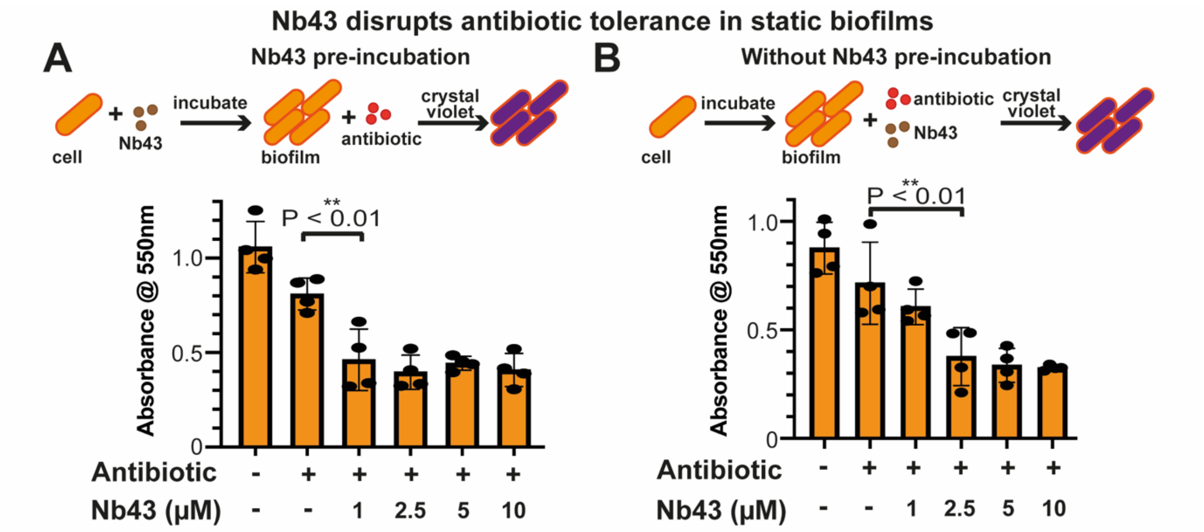

3. The discussion should address the limitations of this therapeutic approach. A key concern is the potential for Pf4 reinfection and subsequent relapse of chronic infection, which is a major challenge in the field. Additionally, the manuscript would be strengthened by a more critical and direct comparison of this Nb-based strategy against existing anti-virulence or anti-biofilm alternatives, highlighting its potential advantages and drawbacks.

We agree that contextualizing the Nb-based strategy, and its limitations and advantages in comparison to other existing treatments, is important. We have added the following sections to the discussion to address these topics.

Discussion, line 311:
Further, the possibility of Pf4 re-infection, due to the rapid exchange of Pf4 within *P. aeruginosa* populations, and relapse into chronic infection is of concern. It is unclear how nanobodies directed against Pf4 phages will affect reintegration and spread of the infection after treatment and should be the subject of further investigation.

Discussion, line 315:

Many strategies are emerging to treat bacterial biofilms, including small molecule compounds or monoclonal antibodies designed to prevent bacterial adhesion, inhibit quorum sensing, along with the use of enzymes to degrade the biofilm extracellular matrix, as well as phage therapy with lytic phages to target bacterial cells in biofilms (53). Furthermore, physical treatments such as light therapy in conjunction with photothermal compounds and ultrasound treatment are being developed (53). Nanobodies have the advantage of high affinity targeting of specific molecules in the biofilm, reducing the risk of off-target effects and potentially the selective pressure for resistance, especially if they are combined with antibiotic treatment. Their size means they are more likely to penetrate into biofilms than monoclonal antibodies, but less likely than small molecule compounds. Other disadvantages include rapid clearance from the body as well as their inherent specificity reducing their target range. These theoretical advantages and disadvantages would need to be exhaustively compared in future studies.

One important advantage of nanobodies is their modularity and amenability to modification which may alleviate some of the disadvantages discussed above. Directed evolution to increase nanobody affinity (54) as well as conjugation to molecules that degrade other matrix components or activate the host immune system could increase nanobody potency in the future (55). Furthermore, since bispecific antibodies targeting the *P. aeruginosa* biofilm matrix component Psl and Type-III secretion system protein PcrV have been tested in the clinic (56), similar multi-specific antibodies could be developed in the future for clinical applications.

Minor comments

1. The prevention of Pf activation in P. aeruginosa biofilms is an important aspect that should be addressed in the Introduction and Discussion.

We have amended the text to cover Pf activation by genetic and environmental factors in the introduction and discussion.

Introduction, line 68:
The activation of Pf4 phage production is stimulated by environmental cues such as oxidative stress (22) and the presence of reactive oxygen and nitrogen species (23), which induce genetic elements that regulate Pf4 such as the excisionase XisF4, which stimulates Pf4 production (24).

2. In the Methods section for the biophysical model, the choice of specific parameters (e.g., phage length a=80 nm, depletant diameter σ=2.4 nm) is justified by referencing the system being modeled. However, a brief sentence explicitly stating that these values were chosen based on the known dimensions of Pf4 and alginate would be helpful for readers that are not familiar with the system.

We have added two sentences to the methods section to explain why these numbers were chosen.

Methods, line 483:
We note that the chosen phage numbers and sizes are not precisely the same as that in the experiments and are smaller to minimize the significant increase in computational complexity and computational cost, however through such choices we

are able to capture comparable phage densities and the role of their (long) aspect ratios (a/b >> 1).

Methods, line 489:
The sizes of the depletant and binder particles were so chosen to efficiently explore, computationally, the role of the depletant interaction, given the phage dimensions.

Significance
This study provides a mechanistic insight into the advance and offers a complementary approach to treating biofilm-related infections, which remains an unexplored area in the field. The reported findings are likely to be of interest and significance to microbiologists and clinicians concerned with biofilm infections.

Thank you.

My own expertise lies in the genetic and biochemical aspects of prophage induction and biofilm formation. Therefore, the details of nanobodies and their potential side effects fall outside the scope of my evaluation.

Reviewer #2 (authors' response in blue):

Evidence, reproducibility and clarity
In the well-written manuscript by Tarafder et al., the authors follow up on their previous investigations of the filamentous bacteriophage Pf4, which self-assembles into a crystalline droplet surrounding Pseudomonas aeruginosa cells within a biofilm. Using theoretical coarse-grained molecular dynamics (MD) simulations, they predict that binding a small molecule or protein to the surface of bacteriophage Pf4 should disrupt the attraction-in this case depletion attraction-between individual phage particles. To test this hypothesis, nanobodies were raised against Pf4, and two promising candidates, Nb43 and Nb-D11, were identified. These nanobodies were characterized using biochemical assays, and binding of Nb43 to CoaB, the major coat protein, was visualized using cryo-EM. Using fluorescence microscopy and cryo-ET, the authors convincingly demonstrate that nanobodies can disrupt Pf4 crystalline droplet formation. Strikingly, nanobody-mediated disruption of Pf4 droplets also increases antibiotic susceptibility of P. aeruginosa both in vitro and in biofilm settings.

We thank the reviewer for this positive summary of the manuscript.

Major comments

1) Theoretical modelling:

The MD simulations, as currently presented, do not add conceptual depth to the study. The idea that blocking an interaction site between phages (whether through active-site interference, obstruction of a protein-protein interface, or simple steric hindrance) would prevent alignment is straightforward and does not necessarily require MD simulations to justify. As such, this section feels superfluous and is currently the weakest point in an otherwise strong manuscript. Unless the simulations can meaningfully address at least some of the questions listed below, the authors should consider removing this part:
The MD simulation is very simplistic, and filamentous phages are clearly not hard rods, as seen in the cryo-EM images. Would a certain degree of Pf4 flexibility allow to stabilize droplets even in the presence of low concentrations of Pf4 binders?

How do the MD simulations explain that already pre-formed crystalline droplets can be penetrated and disassembled by small Pf4 binders?

The authors state that Pf4 binders must be large relative to the depletant particles. Can this be demonstrated experimentally? Is there a sweet spot, as large molecules potentially cannot penetrate preformed droplets?

It is true that, given what we could show experimentally in the rest of the manuscript, the modelling does not alter or change our conclusions concerning successful nanobody treatments of biofilms. However, when performing this study, the modelling was an important first step, which enabled us to commence the experimental work in the first place.

In particular, the modelling allowed us to identify and validate a potential biophysical mechanism by which nanobodies block the depletion interaction between

phages (we note that there is no classical "protein-protein" interface between phages, but rather a lateral association governed by the depletion interaction, which is why blocking the interaction was non-trivial). We agree with the reviewer's comments that we should address modelling assumptions, and we therefore clearly highlight model limitations in the text of the revised manuscript, by making it clear in both the main text and the methods section that our model does not account for charge-based interactions, and only identifies the effect of surface asperities on the depletion interaction.

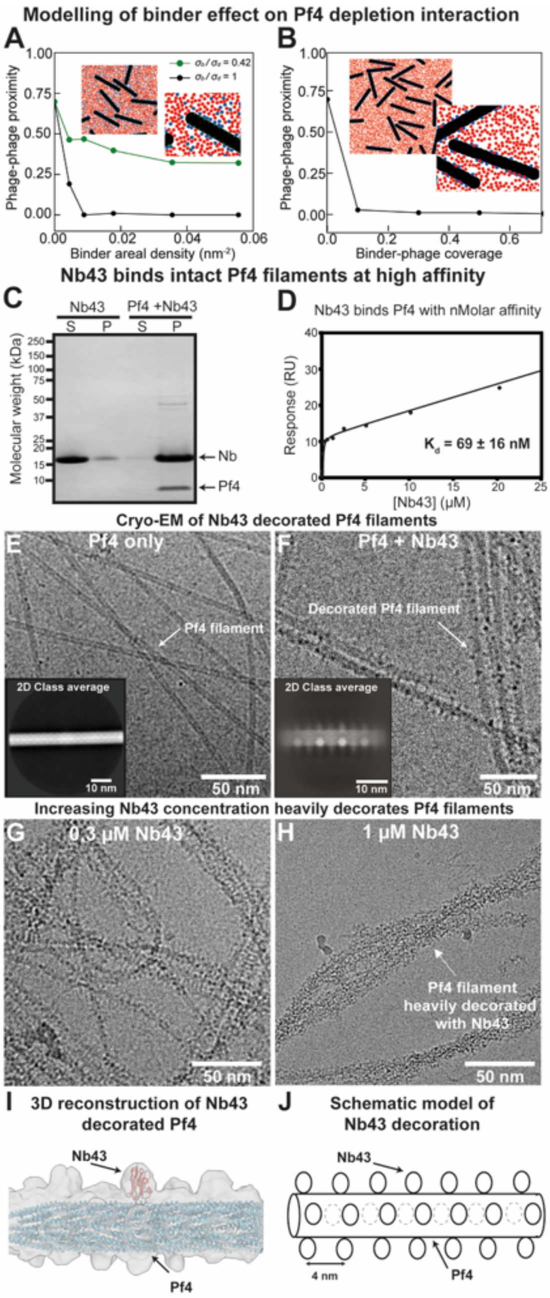

Results, line 127:

In practice, binders may also modify the depletion interaction between phages via charge-based interactions with depletants (see Methods); however, we did not include charge in our simulations, because self-association of phages is generally observed to occur in charge-screened environments that suppress charge-based phage-phage repulsion (21, 31).

Methods, line 545:

In addition to the surface roughening mechanism, the nanobody Nb43 may also reduce the effective size of the depletant, alginate, in the phage-phage depletion interaction. This would involve the phage-bound nanobodies either screening charge-based repulsion between phages and depletants (both alginate and phages are negatively charged) or attracting depletants (the nanobodies are overall positively charged): our simulations do not account for these charge-based interactions. However, because our experiments were performed in a charge-screened environment, it is unlikely that this is a major factor in the nanobody's mechanism. Furthermore, the nanobody's action was found to be replicated in experiments with dextran as a depletant, which carries less overall negative charge (Fig S7A-I).

However, we are keen to take into consideration the reviewer's criticism, therefore we have combined the key finding in the current Figure 1 regarding small binder density (Figure 1E-F) with current Figure 2 to generate a new revised Figure 1 (see adjoining). The rest of the information in current Figure 1 is presented in a new Figure S1. We believe this change will balance the emphasis of the manuscript and address reviewer 2's comments.

2) Nanobody penetration into crystalline droplets (Extended Data Fig. 6a-d) vs. antibiotic penetration (Fig. 4)

The authors show that Nb43 penetrates Pf4 droplets even at concentrations that do not disrupt droplet stability. How do the authors explain that a relatively large nanobody penetrates the crystalline droplet, whereas a much smaller antibiotic does not diffuse trough the droplet?

This is an intriguing question that we cannot fully answer at this point. Our best hypothesis is that the high-affinity binding of Nb43 to Pf4 promotes its retention and association with the droplets, compared to the antibiotic, which does not bind to the droplets with high affinity. We discuss this point in the revised manuscript text.

Discussion, line 352:
Other studies have suggested that Pf4 liquid crystalline droplets protect *P. aeruginosa* by sequestering antibiotics via charge-based attraction rather than a diffusion block (21). This mechanism also relies on Pf4 forming liquid crystalline droplets and would be disrupted by our nanobodies. Interestingly from our light microscopy we do see that Nb43 can penetrate liquid crystalline droplets whereas fluorescent antibiotic does not, presumably due to the high affinity of Nb43 for Pf4 retaining the nanobody within the droplet. Further mechanistic studies on the nature of the diffusion block will be important to clarify the exact mechanism of protection.

In the experiments shown in Figure 4, the authors assess antibiotic activity against P. aeruginosa in the presence of Pf4 crystalline droplets. If I understand correctly, the additionally added Pf4 droplets do not physically encompass the bacteria, yet they still reduce antibiotic tolerance. If so, this appears to contradict the conclusion that Pf4 droplets act primarily as a diffusion barrier (as stated in the section title). Instead, this would suggest that Pf4 may reduce antibiotic potency through another mechanism (e.g., direct binding or sequestration).

Our apologies for not being clearer here. The added Pf4 liquid crystalline droplets encapsulate bacterial cells in the assay presented in Figure 3. We have added a schematic in the revised manuscript to make this clear for readers (Figure 3).

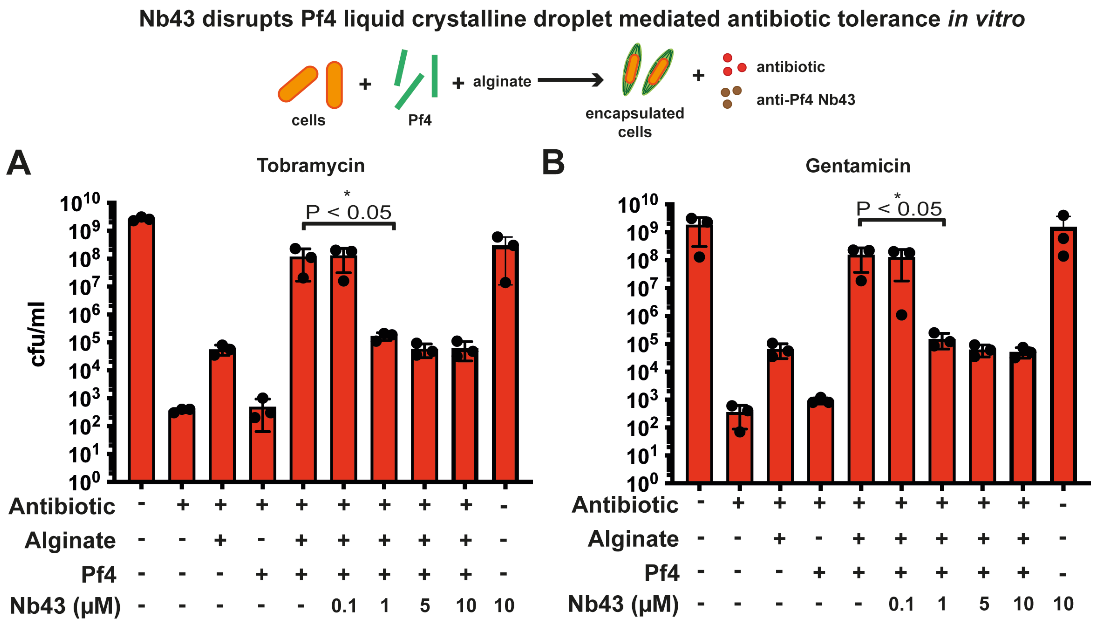

We add the accompanying text to further clarify this point:

Results, line 230:
We have previously shown addition of Pf4 liquid crystalline droplets (i.e., Pf4 with alginate) was sufficient to encapsulate and protect *P. aeruginosa* cells from antibiotic killing *in vitro* (31).

We also discuss alternative mechanisms in the discussion, which may partly explain our observations, and which could be the subject of future mechanistic studies.

Discussion, line 352:
Other studies have suggested that Pf4 liquid crystalline droplets protect *P. aeruginosa* by sequestering antibiotics via charge-based attraction rather than a diffusion block (21). This mechanism also relies on Pf4 forming liquid crystalline droplets and would be disrupted by our nanobodies. Interestingly from our light microscopy we do see that Nb43 can penetrate liquid crystalline droplets whereas fluorescent antibiotic does not, presumably due to the high affinity of Nb43 for Pf4 retaining the nanobody within the droplet. Further mechanistic studies on the nature of the diffusion block will be important to clarify the exact mechanism of protection.

Would it be possible to test the addition of Pf4 alone, without the biopolymer alginate, to determine whether Pf4 itself is sufficient to increase antibiotic tolerance?

We have added this experimental condition to the assays and find that Pf4 alone does not increase antibiotic tolerance. This is presented in a revised Figure 3.

Nb43 disrupts Pf4 liquid crystalline droplet mediated antibiotic tolerance *in vitro*

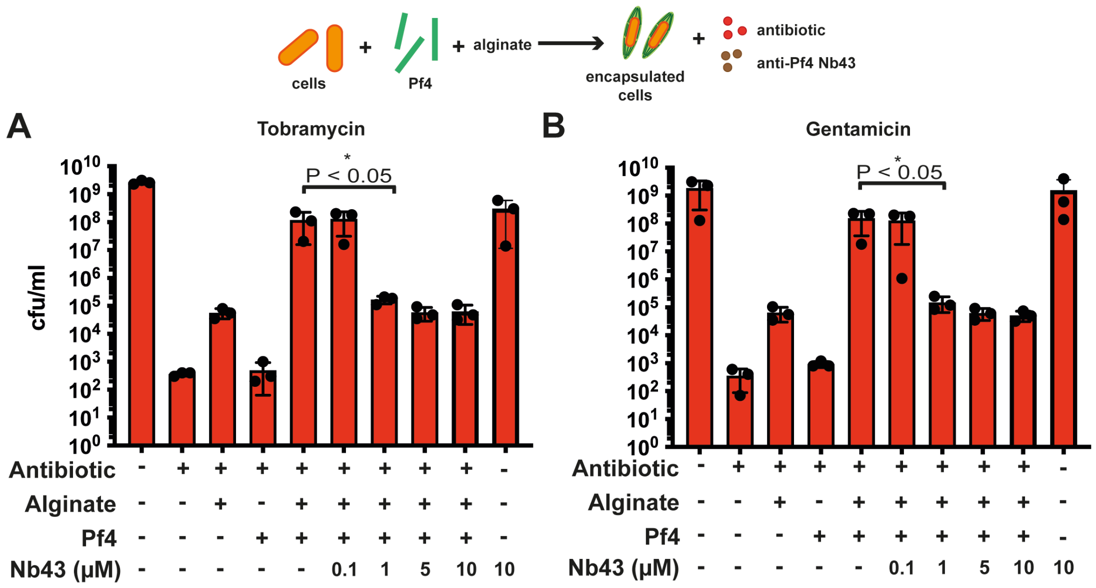

Minor comments:

- Title: The title is overstated. Please consider changing it to something similar to: "Targeted disruption of phage liquid crystalline droplets abolishes antibiotic tolerance in Pseudomonas aeruginosa biofilms."

We have changed the title as requested.
Title, Line 2:
Targeted disruption of phage liquid crystalline droplets abolishes antibiotic tolerance in *Pseudomonas aeruginosa* biofilms

- Introduction sentence:
"...where filamentous phage particles align along their axis in the presence of biopolymer,..."
Please introduce what biopolymers are and specify which types are relevant here.

We have added the following sentence to the introduction to define relevant biopolymers

Introduction, Line 77:
These structures form by depletion attraction interactions, where filamentous phage particles align along their long axis in the presence of host and microbial biopolymers, which are high molecular weight polymeric molecules such as alginate, hyaluronan and extracellular DNA. These biopolymers are excluded from the phage aggregates in the process of liquid crystalline droplet formation.

- Amorphous Pf4 aggregates after Nb43 treatment (Fig 3b,e):

The authors should discuss the nature of these aggregates. It appears that smaller spindles are both broken up and impeded in their formation after Nb43 treatment, whereas larger aggregates seem to persist.

We have added the following sentence to the text.

Results, Line 188:
The treated specimen showed mainly the formation of amorphous aggregates that did not resemble tactoids, suggesting that the ordered formation of spindle-shaped tactoids is impeded by Nb43, but formation of unordered aggregates still persists (Fig 2B).

- Fig. 3c and 3f:
Please describe how liquid crystalline structures were defined in the fluorescence images. Were thresholds for size, intensity, or morphology applied?

We add the following sentence to the methods to clarify:

Methods, line 706:
Maxima corresponding to individual Pf4 liquid crystalline droplets were analysed using MicrobeJ shape analysis functions to quantify area (Huang intensity thresholding, circularity between 0.4-1.0 and size > 0.2 μm^2).

- Use of *P. aeruginosa* ΔPAO728:
For clarity, please explain why the strain lacking the Pf4 integrase is included in the in-vitro assays.

We added a sentence to the results explaining this. Briefly, *P. aeruginosa* ΔPAO728, lacking the Pf4 integrase, does not produce Pf4 phage ensuring the only Pf4 phage present is the purified phage that we have physically added in the assay.

Results, line 234:
We tested this hypothesis by growing *P. aeruginosa* PAO1 Δ*PA0728* (lacking the Pf4 integrase and unable to produce Pf4 phage (21)) in the presence of Pf4 liquid crystalline droplets, nanobody and antibiotic.

-Discussion:
Neisseria meningitidis and Vibrio cholerae use filamentous phages to increase virulence. Do these phages also form liquid crystalline droplets? If not, how do the authors envision that the nanobody strategy described here could be applied to prevent infection? In general, the findings are hard to generalize to other biofilms matrices, which are highly heterogenous.

This is a fair point. All rod-shaped inoviruses will have the propensity to form liquid crystalline structures under the appropriate depletant conditions, but so far they have not been studied in *Neisseria meningitidis* and *Vibrio cholerae* biofilms. Nevertheless, we believe that the strategy adopted to disrupt *P. aeruginosa* Pf aggregates in this manuscript could be applicable to other phages, and potentially even non-phage filamentous molecules that form higher-order structures in the crowded biofilm matrix environment[3]. Examples of such filaments are *B. subtilis* TasA, *E. coli* curli or *P. aeruginosa* Fap fibres. We clarify this point in the discussion section.

Discussion, line 334:

Many bacteria, including *Neisseria meningitidis* and *Vibrio cholerae*, use filamentous phages from the Inovirus genus for increasing their virulence during infection (33, 34, 57). Although all rod-shaped inoviruses have the propensity to form liquid crystalline droplets, it is unclear whether these droplets form in the EPS matrix of these species. However, almost all bacterial biofilms contain a crowded EPS matrix made up of non-phage filaments that self-aggregate into higher-order structures which are key for biofilm integrity and function. Examples of such filaments include *Bacillus subtilis* TasA, *Escherichia coli* curli and *P. aeruginosa* Fap fibres amongst others (12, 35-38), thus similar approaches of disrupting the EPS matrix could be used for biofilm disruption against a variety of targets in different bacteria.

Significance
Bacterial biofilms and their associated antibiotic tolerance represent a major clinical burden, and new strategies to overcome these defenses are urgently needed. The strategy presented here-targeting and disrupting the protective extracellular matrix formed by liquid crystalline Pf4 phage droplets-is an exciting and innovative approach with clear translational potential for combating P. aeruginosa biofilms. The study is experimentally rigorous, well written, and carefully analyzed, and it represents a logical and impactful next step following the group's previous work. This manuscript will have significant impact on the field of P. aeruginosa biofilm research by providing a mechanistically grounded method to disrupt the protective biofilm architecture. However, it is important to note that the extracellular matrix architecture of biofilms formed by other bacterial species differs substantially, and thus the current findings cannot be directly generalized beyond P. aeruginosa without further investigation.

Thank you.
