# Supplementary material for: Disrupting phage liquid crystalline droplets restores antibiotic susceptibility in Pseudomonas aeruginosa biofilms
Source: PLoS Biol. 2026 Jun 5;24(6):e3003834. doi: 10.1371/journal.pbio.3003834 (PMC13262939; doi:10.1371/journal.pbio.3003834)
Supplement: Attachment — Submitted filename: Response_Tarafder_etal.pdf [file pbio.3003834.s018.pdf]

Response to Reviewers

Manuscript number: PBIOLOGY-D-26-00139R2 (RC-2025-03209)
Corresponding author(s): Abul, Tarafder and Tanmay, Bharat

We thank the reviewers for their positive appraisal of our revised manuscript. Please find below our response to the minor request from Reviewer 2.

Reviewer #1: (author's response in blue)

I've re-evaluated the revised manuscript (PBIOLOGY-D-26-00139R2) and the authors' point-by-point responses to all reviewers' comments. I'm satisfied that they've addressed these concerns appropriately. I have no further requests and the manuscript is now ready for publication in PLOS Biology.

Thank you

Reviewer #2: (author's response in blue)

In their revised manuscript, the authors have carefully and thoroughly addressed all my previous comments and I would like to congratulate them on an exciting manuscript.

Just one minor note:
The schematics in Fig. 3A and 4A,B greatly facilitate understanding of the experimental setup. However, it took me a moment to realize that in Fig. 3A the authors used a P. aeruginosa strain deficient in Pf4 production (with Pf4 added exogenously), whereas in the experiments shown in Fig. 4 they used a strain that produces its own Pf4 droplets. It would be helpful if the authors could clarify this distinction in the text and potentially include Pf4 droplets in the schematic of Fig. 4A,B to improve clarity.

We have amended the schematic in Figure 4 to include Pf4 liquid crystalline droplets:

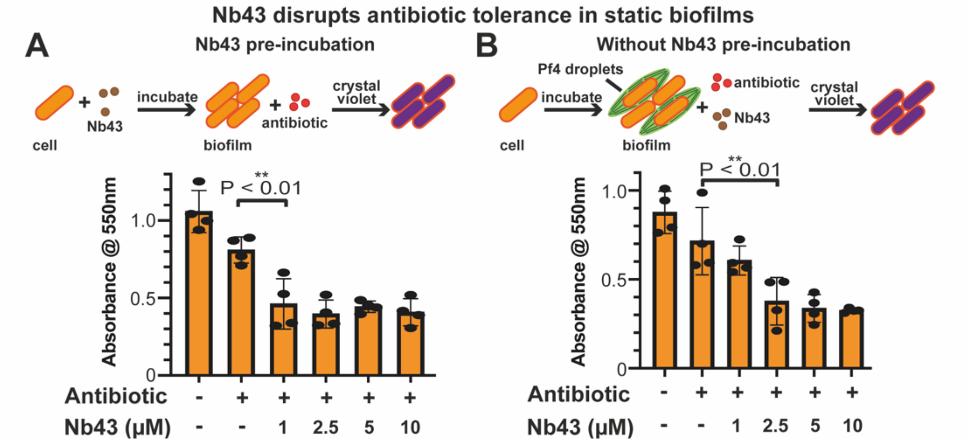

We have also amended the text in the results section to further clarify this point.

Results, lines 261-263:
Given that Nb43 could affect Pf4 liquid crystalline droplet formation, antibiotic tolerance and antibiotic diffusion *in vitro*, we next tested whether this nanobody could affect antibiotic susceptibility of wild-type *P. aeruginosa* PAO1 biofilms producing endogenous Pf4.

We have amended the figure legends of figure 3 and figure 4 to highlight the schematics -

Figure 3 legend, line 425:
A schematic for the experiment is shown at the top.

Figure 4 legend, line 448-451:
(**A-B**) *P. aeruginosa* PAO1 static biofilms were grown in 96-well plates and treated with Nb43 either (A) at the inoculation stage (pre-incubation) or (B) after 24 hours growth (without pre-incubation) with the indicated concentrations of Nb43 (schematics of each experiment shown at top).
